# Adding LLMs to the psycholinguistic norming toolbox: A practical guide to getting the most out of human ratings

**DOI:** 10.3758/s13428-026-03129-3

**Published:** 2026-07-27

**Authors:** Javier Conde, María Grandury, Tairan Fu, Carlos Arriaga, Gonzalo Martínez, Thomas Clark, Sean Trott, Clarence Gerald Green, Pedro Reviriego, Marc Brysbaert

**Affiliations:** 1 Information Processing and Telecommunications Center (IPTC), Universidad Politécnica de Madrid, 28040 Madrid, Spain; 2 Politecnico di Milano, Milan, Italy; 3 Massachusetts Institute of Technology, Cambridge, MA USA; 4 Department of Psychology, Rutgers University – Newark, Newark, NJ USA; 5 Faculty of Education, University of Hong Kong, Hong Kong, Hong Kong; 6 Department of Experimental Psychology, Ghent University, Ghent, Belgium

**Keywords:** LLMs, Word features, Fine-tuning, Tutorial

## Abstract

Word-level psycholinguistic norms are necessary to test theories of language processing. However, obtaining such human-based measures is not always feasible or straightforward. One promising approach is to augment human norming datasets by using large language models (LLMs) to predict these characteristics directly, a practice that is rapidly gaining popularity in psycholinguistics and cognitive science. However, the novelty of this approach (and the relative inscrutability of LLMs) necessitates the adoption of rigorous methodologies. We discuss the range of possible approaches, and clarify limitations that are not immediately apparent. In this work, we present a comprehensive methodology for estimating word characteristics with LLMs, enriched with practical advice and lessons learned from our own experience. Our approach covers both the direct use of base LLMs and the fine-tuning of models, an alternative that can yield substantial performance gains in certain scenarios. A major emphasis in the guide is the need to validate LLM-generated data, at least with a small set of a few hundred human “gold standard” norms, before using the LLM-generated norms. We also present a software framework that implements our methodology and supports both commercial and open-weight models. We illustrate the proposed approach with a case study on estimating word familiarity in English. Using base models, we achieved a Spearman correlation of 0.8 with human ratings, which increased to 0.9 when employing fine-tuned models. This methodology, framework, and set of best practices can serve as a reference for future research on leveraging LLMs for psycholinguistic and lexical studies.

**Supplementary Information:**

The online version contains supplementary material available at https://doi.org/10.3758/s13428-026-03129-3.

## Introduction

The rapid growth of large language models (LLMs) has provided scientists with a range of new tools. In psycholinguistics specifically, one of the most important applications of LLMs has been the estimation of surprisal, the degree to which a word is expected based on the preceding context. LLM-based estimates of surprisal have provided more informative estimates of word predictability (Cevoli et al., 2022; Wilcox et al., 2023), outperforming human sentence completions (known as cloze probabilities; Staub, 2025).

This article discusses the generative capacity of LLMs: the creation of new, artificial data to simulate human performance without having to collect data directly from people (Binz et al., 2025; Long et al., 2024). This generative capacity has been used, for instance, for the generation of synthetic data that can be used to train new versions of LLMs. Evidence suggests that the generative capabilities can be used for other purposes as well, such as augmenting human datasets for scientific investigation. LLMs provide useful (albeit imperfect) estimates for various lexical properties with simple prompts, which would otherwise require expensive data collection from humans.

A limitation of LLMs for psycholinguistics is their lack of explainability (Jain & Wallace, 2019). This is due primarily to the architecture of the models, which are based on complex neural networks, making it very difficult to understand how the model arrives at its output. In addition, the best-performing models currently available are commercial models that are not open to scientific scrutiny (Bommasani et al., 2024). As a result, LLMs are a kind of “black box” that provides intriguing and potentially useful information, without users knowing how the output is generated. There are already studies on the use of LLMs to predict human behavior and refine theories which could lead to a better understanding of how LLMs work (Rivière et al., 2025). However, those are outside the scope of this paper, which focuses on the use of LLM to generate psycholinguistic data.

It should be noted that the inscrutability of LLMs is not specific to artificial intelligence (AI). Psycholinguists have been collecting data on human performance for over 150 years without knowing exactly how the human brain generates the data. When researchers ask participants to rate words on familiarity, they are not solely (or even primarily) interested in how the ratings are made, but whether the ratings are a valid measure of word knowledge and can be used, for example, to predict the difficulty of a text. For many purposes (such as norming experimental stimuli), predictive power is as important as being able to explain the underlying processes (Yarkoni & Westfall, 2017). At the same time, if a researcher’s interest is obtaining human data, human responses are at least (definitionally) *face valid* measures; the same is not necessarily true of LLM-generated responses, and the inscrutability of LLMs is one factor that makes assessing the validity of synthetic data necessary.

The simplest and most obvious way to validate a set of LLM estimates is to compare them with a sample of human data to see how well the AI-generated data match the human data. If they correlate poorly (e.g., *r* < 0.7), you know that the AI-generated estimates are of little use in replacing human data. This may be because the information is not available in the LLM model you are using, or because you have made a mistake somewhere. On the other hand, if the correlation is high, there is no need to collect further human data. For example, Conde et al. (2026) reported that their AI-generated estimates of the concreteness of German words showed a correlation of > 0.85 with human datasets of several thousand words. In such a situation, it makes little sense to collect human concreteness ratings for the more than 1 million other German words that have not yet been rated.

As in any field of research, collecting psycholinguistic data from LLMs requires knowledge of how best to query the models and what steps to take to obtain the most useful output. Over the past 2 years, we have amassed a considerable amount of implicit knowledge about how to access an LLM (e.g., using an application programming interface instead of a web interface), which prompts to use (prompt engineering), how to optimize estimates with fine-tuning, and how to avoid common pitfalls. In this article, we make this implicit knowledge explicit as a practical guide to using LLMs for psycholinguistic norming. We hope that this guide will be useful to researchers in the field, enhance reproducibility, and spark further discussion and iterative improvements regarding best practices. Further, because the focus of this guide is on *practical usage*, we do not discuss theoretical underpinnings of text-based LLMs (e.g., the distributional semantic hypothesis) in detail, except when relevant for discussing concerns with the validity of LLM-generated data.

This manuscript is divided into two main parts. In the first part, we briefly discuss the nature of LLMs and explain how to generate estimates of word features using LLMs based on best practices, as this has consequences for the type of information that can be obtained and how best to obtain it. In the second part (Appendix A), we present our framework^1^ and a step-by-step tutorial for obtaining norms from LLMs with and without fine-tuning. Finally, Appendix B explores the use of models fine-tuned in one language to produce estimates in a different language, a scheme that can be useful if obtaining human data on the target language is not feasible.

## Large language models

### Introduction to LLMs

The widespread use of generative AI began with the launch of ChatGPT 3.5 in November 2022, based on OpenAI's GPT 3.5 model. GPT stands for Generative Pre-trained Transformer. It is a type of language model developed using a neural network architecture known as a transformer, which enables efficient text processing by capturing relationships between a large set of words. The term “generative” indicates that the model can be used to produce new text, rather than simply processing or classifying existing text. “Pre-trained” means that it has been trained on large amounts of text (in the case of the largest models, on billions or trillions of words). This pre-training allows the model to learn which words (and types of words) are most likely to occur in different contexts, often reflecting underlying syntactic rules or semantic associations (Trott & Bergen, 2023; Jones & Bergen, 2024). Once trained, the model is able to predict tokens (fragments of words).

A word is made up of one or more tokens, with fewer tokens assigned to more frequent words (Arnett et al.,, 2024a). Models work with tokens instead of words because the size of the neural network would be much larger if it had to deal with all possible words and their variations, especially given that most models are multilingual. Training a *tokenizer* (i.e., building the dictionary of tokens) is a preliminary phase before training the models. Token dictionary sizes typically range in the tens or hundreds of thousands and are capable of generating any word in the languages supported by the LLM. As an example, the token dictionary used in Llama3 consists of 128 thousand tokens (Grattafiori et al., 2024). The word “picture” is composed of a single token, but the word “picnic” is composed of two tokens “pic” and “nic.”

Most tokenizers have been designed to optimize English, which makes generating the same text in a different language more expensive, slower, and more energy-consuming, as it requires a larger number of tokens (Petrov et al., 2023), although over successive generations, this limitation has been reduced.^2^ Moreover, there is some evidence that tokenization strategies are biased toward certain kinds of morphological and orthographic systems (Arnett & Bergen, 2024; Arnett et al., 2024b).

An English bias is also present for pre-training, where the prevalence of English datasets leads to higher quality in that language (Huang et al., 2025). Some studies even show that models may “translate” input tokens to English in intermediate layers for processing (Wendler et al., 2024). Techniques such as automatic translation of datasets have been used to enhance the training and evaluation of LLMs (Grattafiori et al., 2024; Achiam et al., 2023) to reduce this gap. However, this process shows limitations inherent in the translations themselves (Plaza et al., 2024).

Once a language model has been trained, the output is a set of parameters, mostly weights (hundreds of billions in the largest models), that store the model’s knowledge. These weights are static when the model is used in *inference* mode, meaning that the model does not continue to learn after the training process is complete, but rather applies what has been learned to generate responses or perform tasks.

Although models do not learn while in use, their responses can be guided by information in the *context*. The *context window* refers to the number of tokens that a model can keep track of in a conversation. The context window has increased significantly over the years, from a few thousand tokens to up to 1 million tokens in the Gemini models (Comanici et al., 2025). However, having an extensive context window does not guarantee that the model will track all information in the context equally accurately, as attention tends to degrade with distance in the context, causing certain parts to receive more attention than others (Comanici et al., 20).5

### Explainability and alignment of LLMs

Although the LLM produces output by predicting tokens, the output is not just a single token, but rather a probability distribution over all tokens in the dictionary. The final selection of the output token is made through a weighted sampling based on the probability assigned to each token. One option is to always select the token with the highest probability, making the model deterministic since it (almost^3^) always produces the same response to the same input. However, the typical configuration of the models introduces randomness, which allows for more varied and natural responses, something fundamental to simulating the flexibility of human language. (Note, however, that while introducing randomness does increase the *novelty* of generated text, this does not necessarily reflect increased “creativity”; Peeperkorn et al., 2024).

The probability distribution of the output tokens is expressed as log-probabilities, or logprobs. In principle, with sufficient computational resources, one could produce a high-probability string of length *n* by exploring every possible combination of token sequences in the model’s vocabulary. However, the high number of combinations at each step makes this task computationally infeasible, turning explainability into one of the greatest current challenges of LLMs.

Explainability consists of understanding how all the model’s parameters interact to generate coherent linguistic behavior. One central challenge to explaining or interpreting LLMs is the sheer size of the models: with billions or even hundreds of billions of parameters—and infinite numbers of possible input sequences—identifying the mapping between each parameter and each kind of input is computationally intractable. Researchers have attempted to gain purchase on this question by designing targeted experiments that allow them to probe the inner workings of the models in response to some experimental manipulation (Bricken et al. 2023, Templeton et al., 2024), though interpretability remains an open challenge.

It may be tempting for users to ask an LLM how it arrived at its output. Unfortunately, this is not necessarily an effective strategy (Hu & Levy, 2023). LLMs may not be capable of introspection on the mechanisms that give rise to their own behavior (Song et al., 2025); in general, the fidelity of a model’s explanation for its behavior remains an open question (Turpin et al., 2023). In many cases, an LLM might give a textbook example of what it should take into account, regardless of what is happening within the model.^4^

Since explainability is currently an unsolved problem, one of the key goals in LLM development is ensuring alignment with human interaction patterns. This alignment involves producing responses that are not only grammatical and coherent, but also perceived as useful, safe, and socially acceptable. Ensuring alignment is important to prevent LLMs from negatively interfering with people’s cognitive abilities (Niu et al., 2024).

The lack of explainability, combined with the presence of dependencies and randomness in LLMs, means that output can vary greatly from session to session, depending on how the AI model is configured and how it is handled. This requires a systematic approach to eliciting responses if LLMs are to be incorporated into the psycholinguistic toolkit.

## Obtaining outputs from large language models

There are (roughly) two approaches to eliciting responses from an LLM: through web interfaces or via application programming interfaces (APIs). The choice of approach has implications for control, functionality, and customization.

Web interfaces include chatbots that are accessible via browsers. They are designed for direct communication with users and enable natural conversations. Modern chatbots are not limited to text, but also support audio, images, or video and can generate output in any of these formats. The main advantage is that they are extremely easy and intuitive to use. For example, you can ask a browser to estimate the familiarity of a series of words.^5^

A limitation of most popular web interfaces is that users often cannot adjust the parameters used to elicit text from the model. This has two consequences. First, the output is likely to vary slightly from session to session (see discussion of temperature below), and second, the estimates will be influenced by the questions asked and answers given before (due to the window in which the LLM operates). The estimates are therefore likely to differ if the same word is presented in different word sequences. Another limitation is that ease of use becomes a burden when several thousand or millions of prompts have to be entered manually. Finally, many models hosted on a chat interface have been given a “system prompt” that also influences their behavior, which may not be publicly available (see discussion of closed vs. open models in the following section).

Interaction with LLMs is also possible via APIs, which allow you to communicate with the models using programming languages. This requires knowledge of (the basics of) a programming language (such as Python or R) and the purchase of an API key. API access is generally managed via a pay-per-use model, with costs depending on the number of input and output tokens. With an API, you can retrieve the information from your computer code. This not only automates the search for information, but also allows you to change the configuration of the model parameters, which directly affects the behavior of the model. These settings allow you to influence the style, coherence, or creativity of the generated responses. Among the most commonly used configurable parameters are temperature and top-k.

Temperature controls the degree of randomness in the responses by changing how tokens are sampled from a model’s probability distribution over possible completions in a given context. Low temperature values make the sampling strategy more deterministic and predictable, while higher values yield more varied and unpredictable responses. Ideally, a temperature of 0 should make the system completely predictable, but this is not the case for many commercially available models, because arithmetic operations are implemented on graphics processing units (GPUs) with finite precision, and the order of the operations can lead to slightly different results (Fu et al., 2026). However, the output of two queries with temperature 0 will usually be highly correlated.

The top-k parameter limits the number of options (output tokens) that the model considers when performing the weighted sampling. When top-k = 1, the model always chooses the single most likely answer. To produce norms, top-k is typically set to 1 or the result is the weighted mean of the possible answers based on the logprobs.

There are other parameters, such as top-p, frequency penalty, or presence penalty, that can be used to influence the model’s choice for the next token. So far, we have seen little use in manipulating them. Top-p has a similar effect to top-k, but limits the number of options to those that together form the probability *p*. Frequency penalty and presence penalty are used to prevent token repetition, which is not relevant if the searches are independent of each other. Therefore, we have not investigated whether it makes sense to change these parameters from their default values, although we do not rule out that this might happen in the future.

A final, very important parameter for generating information is the extent to which the model is influenced by previous inputs. If stable estimates are required, you must ensure that each elicitation is a new, independent query. This limits the possibilities for working with batch tests (i.e., giving the model a list of words and asking it to generate an estimate for each of the words). A good way to test whether the estimates are influenced by previous estimates is to use two different permutations of the list for which you need estimates. In our experiments we have found that if the two estimates correlate less than 0.96, there are likely dependencies in the estimates that are obtained (or the range of estimates is likely too small to obtain reliable differences in estimates). This is a guideline rather than a fixed number, as the exact value of the correlation may depend on the model implementation and the impact of the nondeterminism of GPU execution previously discussed (Fu et al., 2026).

## Which LLM to use?

There is a large and rapidly growing variety of LLMs to choose from, many of which can be found at companies’ websites (for commercial models) or public repositories such as Hugging Face^6^ (for open-weight models).

The first selection criterion is whether the LLM is commercial, open-weight, or research-based. **Commercial** models do not share the model weights (or the training data), so all interactions must take place via the model owner's servers. Examples of this type are the GPT or Gemini model series from OpenAI and Google. **Open-weight** models, on the other hand, do publish the weights, allowing people to download the model and run it in inference mode using public or private compute infrastructure (which is not that of the model owner). Although the weights are accessible, the training data and full details of the development process remain hidden. Models such as LLaMA (Meta) or DeepSeek (DeepSeek AI) are examples of this category. **Fully open** models give users complete control over the training material and training regime. Platforms and models such as Pythia (Biderman et al., 2023), OLMo 2,^7^ or Apertus^8^ provide a suite of LLMs with all the data and process public to be used in interpretability experiments. Other advantages include that they are free to use and that one can study (and manipulate) the model's operation. The main disadvantage of these models at present is that they do not achieve the performance level of commercial and closed-weight LLMs. At the same time, a chief concern with proprietary models (as well as open-weight models, for which the training data are unknown) is *data contamination*: the possibility that they have been trained directly on data used to evaluate the model. This makes evaluating the capabilities of those models extremely difficult (if not impossible). Another major limitation of commercial models specifically is reproducibility: because much of the model workings are hidden behind an API, their behavior may vary across repeated experiments, making it impossible to *exactly reproduce* their behavior. See additional work (Liesenfeld et al., 2024; Hussain et al., 2024) for a much more detailed consideration of the concerns relating to commercial, open-weight, and fully open models.

The second selection criterion is the quality of the model. If the primary aim is to obtain and publish psycholinguistic norms that approximate human judgments, the performance of the model may outweigh concerns about openness and justify the use of a closed-source, proprietary model. However, if differences between models are small and have little practical value, other considerations come into play, such as the increasing commitment in psycholinguistics to open science and inclusive practices. At present, and from our experience in these tasks, proprietary models offer significantly better performance, but as research progresses, we hope that open-source alternatives can be prioritized. Cost–benefit considerations matter for the use of public research funds, as does the relationship between public funds and private enterprises. If an open-source model yields correlations within the acceptable range with human norms, we recommend the use of that model for the reasons stated above (reducing the possibility of data contamination and allowing for direct, exact reproducibility).

A typical approach to evaluating different models is comparing their output to validation datasets (Conde et al., 2025). A validation dataset consists of human data for the variable of interest. For example, if one wants to obtain LLM estimates of the age at which a word was learned (age of acquisition, or AoA), one looks for a set of human AoA ratings with which to compare the output of the LLM (Hinojosa et al., 2016). Ideally, multiple human sets are available so that one can see not only how well the LLM output correlates with the human ratings, but also to what extent this correlation is comparable to the correlations between different sets of human ratings, i.e., *inter-annotator agreement*. An inter-annotator correlation score of about 0.8 or 0.9 is generally considered reliable. Moreover, the degree of inter-human correlation represents a threshold for humanlike model performance: if a model produces values for a particular psycholinguistic norm whose correlation with human data is indistinguishable from the inter-human correlation, it suggests that individual model judgments may be substitutable for individual human judgments, in the sense that replacing a random human judgment with a model judgment would not meaningfully change the average judgment for that item. In some cases, model outputs have shown even higher correlations with the human average than have individual human judgments, suggesting that model judgments may capture a partial “wisdom of the crowd” (Trott, 2024b).

If no human ratings are available for a domain of interest, we strongly recommend collecting them for a representative sample of at least several hundred words. This allows researchers to perform the necessary validation checks. An additional advantage is that the human ratings have not yet influenced the LLM estimates, i.e., there is no possibility of direct contamination given that the ratings are entirely new. As noted previously, one concern with LLMs (especially those whose training data are not publicly available) is that their output can be influenced by datasets available on the internet. In these cases, the LLM might produce good estimates for words in the dataset on the internet, but not for new words. When validating the output of an LLM on an established dataset, one must indeed always take into account that good performance on that dataset cannot necessarily be generalized to other words. Therefore, it is always a good idea to perform some additional checks on the quality of the estimates obtained with words that are not yet available in the literature. **In general, we cannot recommend publishing LLM-generated norms if the methodological procedure for generating those norms has not been validated with human data**.

Human ratings are now easier to collect than in the past. For most ratings, we see that participants give approximately 1,000 ratings per hour. These ratings can be completed online. Furthermore, most ratings are reliable (*r* > 0.9) once 10–15 participants have independently rated the stimuli. There are therefore few objections to collecting a representative validation set of 1,000 ratings, which you can use to obtain AI-generated estimates for all other words you are interested in. When collecting new data, researchers must clearly inform participants when their data will be used to train or fine-tune AI models. Participants should have the option to decline this use without any impact on their involvement in the study, ensuring transparency and respecting their autonomy.

Until 2025, we found that GPT-4o outperformed other models tested on the datasets we explored (constituting a range of languages and linguistic constructs), but this may not remain the case: other models are likely to improve, new models will be introduced, and the company developing GPT may make changes that make the model less suitable for estimating psycholinguistic word characteristics. Further, any given model can be “improved” by fine-tuning that model on a pool of existing psycholinguistic norms (see below).

The best way to ensure that you are working with a good model and that you have done everything correctly is to compare the model's output with (new) human data. This should be a basic check when using LLM estimates. Beyond correlating judgments with a new gold standard, researchers can conduct *substitution analyses* to ask whether LLM-generated norms provide equivalent predictive validity, e.g., of reaction time or other dependent variables of interest (Trott, 2024a). Such analyses might also inform questions about the construct validity of LLM-generated norms: if synthetic norms correlate strongly with human norms, *and* they predict other variables of interest in similar ways as do human norms, it suggests they might serve a useful function for psycholinguists. Researchers can also investigate LLM-generated norms for the presence of *systematic biases*. For example, Trott (2024a) found that GPT-4 systematically overrated the similarity of antonyms and also exhibited particularly poor performance when rating the similarity of *concrete* (as opposed to *abstract*) word pairs. In some cases, the presence of systematic errors (i.e., relative to human judgments) may suggest that the mechanisms underlying LLM-generated judgments are different in important ways from those underlying human judgments. Thus, while the direct correlation with human ratings is probably the most important indicator of suitability, other analyses may help researchers triangulate the question of whether LLM-generated judgments are appropriate to use in a given context.

## Prompt engineering

The next decision that will affect the quality of your LLM estimates is the prompt you use to elicit a given output. Some prompts are more effective at eliciting human-aligned judgments than others. Finding the best prompt is called *prompt engineering*. Prompt engineering involves selecting the target language, context, instructions, and so on.

Prompt engineering is an active research area, but there is evidence that small variations in prompts can produce different responses. For example, specifying the desired tone (“write in a formal style”), indicating the output format (“return a numbered list”), or setting the model’s role (“act as a language expert”) could affect the quality of the response. One widely used and seemingly reliable technique is called few-shot prompting, in which a model is provided with one or more examples of the task; typically, these examples help the model produce answers in the appropriate format that are more aligned with the user’s goals.

When thinking of a good prompt, it is wise to keep in mind that LLMs are trained to produce the most likely next token. Ideally, a good prompt should point the model directly to that token. For instance, we found that the following prompt worked well producing concreteness estimates for words that correlated better with human estimates (Martínez et al., 2024):

Could you please rate the concreteness of the following word on a scale from 1 to 5, where 1 means very abstract and 5 means very concrete? Examples of words that would get a rating of 1 are essentialness, although, and hope. Examples of words that would get a rating of 5 are bat, frangipane, and blackbird. The word is: [insert word here]. Only answer a number from 1 to 5. Please limit your answer to numbers.

This prompt directly asks for concreteness estimates between 1 and 5; it explains what 1 stands for and what 5 stands for. However, it does assume that the model “understands” concreteness, as no definition is given. When it is unclear whether a model will understand the construct in question, we recommend giving an explicit definition of the construct in the prompt, e.g., by starting the prompt with the sentence “Concreteness is a measure of how concrete or abstract something is. A word is concrete if it represents something that exists in a definite physical form in the real world. In contrast, a word is abstract if it represents more of a concept or idea” (Scott et al., 2019).

The prompt we provided also includes three examples of words that would be given an estimate of 1 or an estimate of 5, which help scaffold its responses. When these are not given, the model is left on its own to decide what the extremes are. Prompting without examples is called zero-shot prompting. In the prompt above, we used three-shot prompting. Further instances could be added (e.g., five-shot prompting) if that improves performance.

The advantage of zero-shot prompting is that you rely entirely on the LLM's pre-existing knowledge and its understanding of natural language instructions. You do not influence the output in any way. The disadvantage is that the model may work with suboptimal choices, for instance using too narrow a range, so that the estimates are squeezed between 2 and 4. The only way to see whether giving examples is helpful is to compare the output of the different prompts to a set of human ratings (or other information you have about the variable).

The prompt we used also instructs the model to provide only numbers. This is necessary because otherwise the model may produce irrelevant output (e.g., comments on the given word or on the task in general). Even with explicit instructions, it is necessary to always check the output for incidental hallucinations, i.e., responses that are not to be expected based on pragmatic human conversation.

As explained in the previous section, we repeat the prompt for each word in an entirely new API call to avoid the risk of dependencies in the answers. Nevertheless, it is good practice to occasionally check that the estimates do not differ depending on the order in which the words are given within a session, and that an estimate is given for each word.

Occasionally, we have observed that the LLM does not provide an estimate for a word (e.g., a taboo word or even a random word from the list) and immediately moves on to the next word. This carries the risk that the words and estimates will not match if the pipeline is fully automated without cross-checks. One way to protect yourself against misalignment is to ask the model to provide both the word and the estimate by giving the command “The output format must be a JSON object. For example: {"Word": "{word}", " Concreteness"://Concreteness of the word expressed as a number from 1 to 5}.” This makes it easy to check whether estimates have been provided for all words and, if not, which words are missing. However, this can have disadvantages. In general (but not always), we have found that asking for additional information carries the risk of reducing the quality of the critical estimate, as there is no longer a direct link between the prompt and the output. When asking for additional information, it is a good idea to check that this does not affect the answers given.

The prompt we used was in English. An open question is what the best prompt is for other languages. A natural intuition is that prompts in the target language (Spanish, German, Chinese, etc.) would be better, as this increases the connection between the input and the desired output. However, we have occasionally observed that the output is better when the prompt is in English, even to obtain estimates for another language. One way to understand this is that the LLM has to rely on its prior training to interpret the prompt correctly (what do the words concreteness, essentialness, bat mean?). If a model is not well trained in the target language, it is possible that an English prompt cues more activations that are relevant to the construct of interest than a prompt in the target language. The only way to determine whether this is the case is to, again, compare the output of the different prompts with a validation set.

A final point is the granularity of the output. In our prompt, we requested a single number between 1 and 5. One could argue that this output is too coarse to be useful and that a finer distinction is desirable. One way to achieve this is to increase the range of numbers (e.g., between 1 and 100) or to request output with decimals (e.g., two decimals). This may improve the fidelity of estimates, but increased fidelity is not guaranteed: we have noticed that the model sometimes only produces a subset of possible answers. For example, in a recent study generating AoA for English words, GPT-4o tended to prefer whole number estimates, even when instructed that it could include decimal places and the model was fine-tuned to 2,000 ratings of Kuperman et al. (2012) that included decimal places. One factor that could influence a model’s behavior here is the number of tokens associated with a given rating: generally, a number with decimals (4.25) will consist of more tokens than a whole number (4).^9^ Therefore, using an extended range of integers is usually better.

A better alternative, when it is available, is directly inspecting the probability associated with different answers. If the logarithmic probabilities (logprobs) associated with different tokens are available, researchers can calculate a weighted average using those logprobs. For example, a model’s most likely estimate for the familiarity of zucchini may be 4 (out of 5), but the logprobs may indicate that the probability of answer 3 is 0.1, that of answer 4 is 0.5, and that of answer 5 is 0.4. The weighted average is then 0.12 × 3 + 0.45 × 4 + 0.43 × 5 = 4.31. In this way, we have calculated estimates of word familiarity, concreteness, valence, and arousal (Conde et al., 2025; Brysbaert et al., 2025). However, this does not always work when fine-tuning is applied, as we will see below. With an open-weight model provided through platforms like HuggingFace, the probability associated with any arbitrary token completion can be obtained, allowing researchers to obtain a probability distribution over the entire space of possible completions (if desired); in contrast, proprietary models in most cases only provide access to the top-k tokens, and in the case of OpenAI, typically the top-5 or top-20 options.^10^ This is yet another advantage of (relatively) open models.

## Fine-tuning

LLMs are trained to predict the next token based on an input sequence. However, their popularity has grown with the emergence of chatbots. Chatbots do more than conveying information from the base model directly. They have been retrained within a second phase known as instruction-tuning. In this phase, the weights of the LLM are adjusted using specific datasets containing examples of questions and answers, allowing the model to adapt its behavior toward conversational interaction.

Fine-tuning is particularly effective in the final stages of model development, when all the knowledge from pretraining has already been acquired. At this point, the goal is not to learn new facts, but to adapt its output to specific tasks, modify its response style, adjust its tone, avoid harmful responses, and more. This personalization is possible because the process directly modifies some of the model’s weights, so that the model becomes tailored to a specific task. Although fine-tuning alters some of the model’s parameters, its ability to incorporate new knowledge is limited. Knowledge acquisition occurs primarily during the early stages of pretraining, when the model is exposed to vast amounts of text. Fine-tuning, by contrast, does not aim to expand general knowledge but to fine-tune the model’s output for specific contexts or tasks.

The best-known example of fine-tuning is that of conversational models, but it is not the only one. Today, there are many other uses of the technique. For example, reasoning models are developed through an additional fine-tuning phase in which reinforcement learning with human feedback (RLHF) is applied, training the model to explore effective reasoning sequences. Fine-tuning can also be used to adapt a base model to specific tasks such as sentiment classification, summarization, or entity extraction. In these cases, the model becomes more competent at specific tasks thanks to the refinement of its behavior based on representative examples.

Our recent research suggests that fine-tuning can also be used to provide psycholinguistic estimates that are more in line with human judgment. Here, a model can be fine-tuned on a limited training set of words with human judgments, allowing researchers to ask whether this fine-tuning improves the quality of generated estimates for other words not in that fine-tuning set. For instance, Sendín et al. (2025) found that AoA estimates for Spanish words did not correlate as strongly with human judgments as the human judgments did with each other. The authors refined the model based on 2,000 human ratings and found that the correlation with human ratings increased to the same level as the correlations between the human ratings, even for words not included in the fine-tuning set. This suggests that fine-tuning may be particularly useful for languages that are underrepresented in the model's training or for response types that may be less “natural” to produce (like response time).

While fine-tuning should always be considered, some data indicate that it may be less helpful for some languages (like English) or tasks. For example, a recent study generating English AoAs investigated a trained model fine-tuned on 2,000 ratings from Kuperman et al. (2012), and although fine-tuning increased correlations with human ratings, there was not much improvement over zero-shot untrained estimates for the same words (Green et al., 2025). This likely reflects the fact that the LLMs are overwhelmingly trained on English language data, or that this dataset is in the training dataset.

Fine-tuning works by first asking the model for an estimate and then providing feedback on the expected number. The feedback is used by the model to adjust the weights so that the output is closer to the expected output. The weights are readjusted sequentially for each word in the fine-tuning set. After fine-tuning, the adjusted model can be saved for future use.

Again, fine-tuning is a non-deterministic technique involving many variables (size of the training dataset, sampling strategy, model configuration, parameters, etc.). It is fair to say that we do not yet have a complete picture of what the best fine-tuning strategy is. How detailed can the feedback be (for example, can we use decimals for ratings from 1 to 5)? What are the most informative stimuli for fine-tuning a model? To what extent can the items we use skew the estimates of the other items? How large should the sample be? What information should the model provide (e.g., word + estimate or estimate only?) and what feedback is given to the model? These are all parameters that may or may not affect the quality of the fine-tuning and do not necessarily have to be the same for all languages and all variables that can be tested.

Fine-tuning of LLMs is a promising tool for generating word features, as it can enable researchers to obtain useful information for an entire language based on a subset of the words, typically a few thousand. In our recent studies (Sendín et al., 2025; Martínez et al., 2025), we indeed found that it was possible to obtain useful response times for English lexical decisions after fine-tuning GPT-4o mini on RTs for 3,000 words collected in a megastudy. Instead of collecting lexical decision times for tens of thousands of words, it is therefore possible to estimate them based on a sample of 3,000 words. Similar results were recently obtained for sensorimotor norms in Wu et al. (2026), suggesting that fine-tuned LLMs are capable of estimating not only lexical features but also behavioral or sensorimotor norms. Therefore, our guidance is to follow the procedures described in this paper and for a given feature evaluate whether LLMs can provide valid estimates even when features are not lexical. The fine-tuning may be particularly interesting for populations that are difficult to reach and therefore cannot be tested in large-scale megastudies (Siew et al., 2025), or to try out different hypotheses to determine which items are likely to yield the most information in tests with humans. The potential of this fine-tuning is yet to be explored, with initial works indicating that fine-tuning does not always yield good results (Kandala & Hoemann, 2025).

Another question is whether fine-tuning in a given language (e.g., English) improves performance in other languages (e.g., Spanish). This is especially important when the target language is relatively underrepresented in a model’s training data. This is typically the case for languages other than English, as in most models the majority of the training data was in English; for example, in GPT-3, more than 90% of the training data was in English (Brown et al., 2020). In our research on familiarity estimates, we observed gradually deteriorating results for zero-shot queries from a model fine-tuned in English to Spanish, German, and Dutch. Future research could attempt to develop *theoretical principles* for when improvements from fine-tuning in one language should be expected to transfer to another language.

## Pitfalls, recommendations, and open questions

Throughout the guide thus far, we have discussed several common pitfalls researchers might encounter when using LLMs to generate psycholinguistic norms. In this section, we expand on these concerns, including our own recommendations for addressing them. We also discuss a number of open questions relating to the validation and use of LLM-generated norms.

### Common pitfalls

First, as noted above, researchers may use approaches that do not allow sufficient experimental control, such as using the web interface for ChatGPT rather than the API; similarly, researchers may fail to *report* the precise parameters used to produce the outputs they analyzed, such as temperature and the exact prompt. Model openness relates to this concern: the use of a commercial model (such as GPT-4) often obscures the specific parameters involved in generating responses, which means that even if researchers report the *controllable* parameters used in their study, other researchers may fail to reproduce the exact responses they obtained. Commercial models also carry a higher risk of being deprecated or replaced by the model creators. Thus, we recommend using fully open models (where possible), as well as reporting all relevant parameters used to elicit responses from an LLM, to maximize the chance of reproducibility by other researchers.

A second pitfall is insufficient validation. As emphasized throughout the paper, we cannot recommend the use of LLM-generated norms when the procedure used for obtaining those norms has not been *validated* using a human “gold standard.” That is, researchers should ensure that the generation process (including the model, temperature, prompt, and construct of interest) results in judgments that align well with a sample of human judgments. Since it is currently feasible to collect ratings for 1,000 stimuli, we recommend that authors aim for this number. A smaller number of stimuli can be justified if they have been carefully selected from across the entire range; more stimuli are better, although the more human data one collects, the less need there remains for AI-generated estimates. We recommend calculating the correlation between human judgments (e.g., the average of multiple independent human judgments) to LLM-generated judgments; human inter-annotator agreement can be used as a baseline for the degree of alignment expected. We also recommend conducting additional analyses, such as the substitution analysis or error analysis described earlier (Trott, 2024a), which can shed light on issues relating to the *validity* of LLM-generated stimuli; this question is addressed in more detail in the following subsection.

Another pitfall is the possibility of *data contamination*. As discussed earlier, data contamination is when the same data are used to train and evaluate a model. Concretely, data contamination raises challenges for validating an LLM’s ability to generate useful psycholinguistic judgments: if an LLM has been trained on concreteness judgments for words like “table” or “freedom,” then its ability to *reproduce* those exact judgments may not reflect is ability to produce judgments for words it has not been trained on. There are several approaches to addressing data contamination when validating LLM-generated norms. First, with fully open models, researchers can conduct an *audit* of the training set to determine whether the data used to evaluate an LLM were included in its training; if they were, a different dataset (i.e., new words with new judgments) should be used. This is (again) an advantage of using fully open models, and a disadvantage to using commercial models. Second, even with commercial models, researchers can use datasets that were extremely *unlikely* to have been included in a model’s training set. The best candidates here would be datasets collected by the researchers themselves. Researchers could also use datasets published after a model’s reported “cutoff date,” though notably, the cutoff date cannot necessarily be validated or verified. Third, researchers can deploy methods developed to detect whether a particular string was present in the training set (Golchin & Surdeanu, 2023). These methods have the advantage of being usable even for commercial models, but their primary disadvantage is that they are imperfect and may yield false negatives.

### Open questions

Using LLMs to generate psycholinguistic norms is a relatively novel approach. Therefore, there are a number of open methodological and theoretical questions relating to their use. The primary purpose of this manuscript is to serve as a practical guide; nonetheless, we briefly touch on some of these issues here.

One notable question is *when* and *how* to integrate LLM-generated norms into the psycholinguistic pipeline (e.g., norming data). At the level of individual researchers, this decision will be informed by both empirical analyses (e.g., correlation with a human gold standard) and theoretical concerns (e.g., face validity; see below). Yet as with many methodological questions, we suspect that individual decisions will depend on field-specific consensus about “best practices,” such as the use of *p *< 0.05 as a threshold for statistical significance. These best practices have yet to be developed. Our recommendation, however, is that LLM-generated norms should currently only be used to *augment* human datasets: that is, researchers should not rely on LLM-generated judgments for stimuli that have not been validated with a sample of human judgments.

This connects to a second open question: namely, whether LLM-generated norms exhibit construct validity. As described in Trott (2024a), there are several approaches to addressing this question. One is theoretical, and corresponds roughly to *face validity*: are LLMs the “kind of system” capable of generating responses about a given construct? Here, some constructs (such as part-of-speech) might seem intuitively like more likely candidates than others (such as moral valence). Details of LLM training procedures might be relevant here as well: for instance, LLMs are not currently trained on olfactory or gustatory inputs, so one might decide that they are not suitable for answering questions about taste or smell, except insofar as a researcher’s goal is determining whether such information can be gleaned from the distributional statistics of language alone. A second approach is empirical, and relies on validating LLM-generated judgments by comparing them to a human gold standard (see above) or asking whether they correlate with *other* data of interest (such as lexical decision times) in similar ways as the human gold standard. Empirical approaches might also be used to address theoretical questions, such as the degree of *alignment* between the mechanisms underpinning LLM-generated and human-generated responses (Pavlick, 2023).

Another set of open questions relates to the *statistical analysis* of LLM-generated data. At present, psycholinguists deploy a number of statistical techniques developed to address common issues relating to experimental design and data collection (e.g., repeated-measures designs). For instance, nonindependence in data (such as multiple responses from the same participant) is typically addressed using mixed effects models that include *random effects* terms intended to account for those sources of nonindependence. It is unclear how to conceptualize potential sources of nonindependence in LLM-generated data. For example, if a researcher uses the same model to produce 100 responses to the same stimulus with the same prompt (i.e., with temperature > 0), how should those responses be analyzed? Intuitively, the researcher could include random effects terms for the model, prompt, and item, under the assumption that this procedure roughly corresponds to eliciting responses from the same person at different points in time, yet this assumption may not be correct, and a given LLM response may be more analogous to the *average* between multiple human responses (Trott, 2024b). Relatedly, most work on LLM-generated norms has focused on their ability to produce responses that correlate with the human average. It is unclear whether they can be used capture *individual differences* in responses, or whether such an approach is even theoretically sensible.

Finally, the use of LLMs in this capacity raises questions about research ethics and the responsible use of technology in research. We have touched on some of these issues already, such as reproducibility. Yet there are also deep philosophical questions about the use of systems like LLMs in psycholinguistics and the potential “epistemic gaps” this introduces into the scientific pipeline (Messeri & Crockett, (2024). This is why our current recommendation is to keep the “human in the loop,” i.e., using LLM-generated norms to *augment* (rather than *replace*) human-generated norms. Further, we note that there is a growing body of literature on the ethical issues relating to the use of AI in scientific practice (Messeri & Crockett, 2024; Agnew et al., 2024; Lin, 2025; Abdurahman et al., 2024; Wang et al., 2025).

## Conclusion and limitations

In this work, we have shown that LLMs can be a useful tool for estimating word characteristics that require human judgments. LLMs can generate large-scale estimates for the 100,000 most interesting words in a language within a few hours and at very low cost. They can thus supplement and expand on human estimates, which are typically limited to a few hundred or thousand words and require much more time and expense. To ensure that LLM estimates match those of humans, we emphasize the importance of checking their validity against a set of human data before using the LLM to generate large-scale estimates. Because of the novelty of these models, their rapid adoption by the scientific community, and the (often overlooked) factors influencing the responses they produce, there is a need for practical guides that explain how to fully exploit their potential, avoid common pitfalls, and maximize the chance of reproducibility by other researchers.

To address this need, we have presented a guide that explores both the direct use of base LLMs and the training of specialized models that leverage the prior linguistic knowledge of these base models. We have provided practical guidelines and insights derived from our experience, including prompting strategies, approaches for evaluating results, model configuration and selection, dataset sampling methods, and fine-tuning. These contributions are accompanied by a framework that we believe can serve as a reference for future research in the field (Appendix A). The use of the framework will make the studies replicable and allow the steps taken to be traced, something crucial in this type of research since, as has been seen, some strategies work very well in certain scenarios and not in others. We have also discussed common pitfalls and open questions relating to the use of LLMs in producing psycholinguistic norms. Appendix A includes a step-by-step guide showing how to execute the framework through the English familiarity with and without fine-tuning using both a commercial model (GPT-4o mini) and an open-weights model (Llama3.1-8B).

Fine-tuning has emerged as a promising technique for improving model alignment with target tasks, although its effectiveness varies depending on the specific lexical feature and language under consideration. Fine-tuning not only improves model performance in certain cases, but also helps reduce the uncertainty associated with the direct use of an LLM, as it tends to be less sensitive to prompting strategies.

Finally, the use of LLM-generated norms has several limitations:

1. **Inscrutability.** LLMs operate as highly complex neural networks with billions of parameters, making their internal reasoning processes effectively opaque. For commercial models, this issue is even more pronounced, as they are provided as black boxes, preventing any inspection of their internal mechanisms, the precise parameters underlying their responses, or the data used to train them.
2. **Uncertainty about optimality**. The results presented are based on months of experimentation with the proposed approach. However, given the lack of interpretability of LLMs, we cannot ensure that our solution is the optimal one. It is likely that future research will introduce alternative strategies that outperform our method.
3. **Dependence on commercial models**. The best performance was achieved with commercial LLMs. This introduces the risk that if these models are discontinued, they will no longer be available for use. Furthermore, there is no guarantee that future versions of LLMs will outperform current ones in psycholinguistic tasks of the kind explored in this paper. Fine-tuning can serve as a strategy to mitigate this risk by retraining models to the specific task.
4. **Variability across languages, characteristics, and models**. We experimented with multiple languages (English, Spanish, German, Dutch), lexical characteristics (familiarity, age of acquisition, valence, arousal, etc.), and models (GPT-4, Gemini 2.5, LLaMA). We found that performance varied considerably across these dimensions. This highlights the importance of having a reliable, human-validated dataset to assess whether model estimations are sufficiently accurate for each specific context.

## Supplementary Information

Below is the link to the electronic supplementary material.

Supplementary file1 (DOCX 3.29 MB)

## Data Availability

All data and materials are available at https://doi.org/10.5281/zenodo.17141353.

## Footnotes

1 https://github.com/WordsGPT/psycholinguistics_framework

2 https://openai.com/index/hello-gpt-4o/

3 Even if the temperature is set to 0 to always select the token with the highest probability and with the same configuration of parameters, small differences may still occur in the output of LLMs: https://152334h.github.io/blog/non-determinism-in-gpt-4/

4 To illustrate, we invite readers to ask an LLM to estimate familiarity for a set of words and then ask the model how it arrived at the estimates.

5 Again, the reader is invited to do so—for example, “Can you give me the familiarity (from 1 = very unfamiliar to 7 = very familiar) of the following words: psycholinguistic, child, aardvark, ambitious, and zucchini?”

6 https://huggingface.co/

7 https://allenai.org/olmo

8 https://ethz.ch/en/news-and-events/eth-news/news/2025/09/press-release-apertus-a-fully-open-transparent-multilingual-language-model.html

9 For example, in the GPT-4o tokenizer, integer numbers from 0 to 999 are represented using a single token. In contrast, decimal numbers are split into several tokens—for example, 4.25 is represented by three tokens: “4”, “.”, “25”. Therefore, instead of using decimal numbers, it is better to use an extended range of integers.

10 https://platform.openai.com/docs/api-reference/completions

## Code availability

The Python framework and code used for running the experiments is available at https://github.com/WordsGPT/psycholinguistics_framework.
