# Supplementary material for: Adding LLMs to the psycholinguistic norming toolbox: A practical guide to getting the most out of human ratings
Source: Behav Res Methods. 2026 Jul 27;58(9):253. doi: 10.3758/s13428-026-03129-3 (PMC13407732; doi:10.3758/s13428-026-03129-3)
Supplement: Supplementary file 1 — Supplementary file1 (DOCX 3.29 MB) [file 13428_2026_3129_MOESM1_ESM.docx]

## Doing the work

Now that we have described the main steps to obtain LLM estimates for psycholinguistic variables, we will elaborate a few examples. In doing so, we will briefly repeat some of the information already given. The reason for this is that we think most readers will either read the first seven sections or this Appendix. They will read the previous sections when they want to get a general idea, and they will want to dive directly into this section when they want to apply the methods to a specific question.

Here, we present a framework and define a methodology based on our experience in the past two years, when we developed techniques for a range of studies involving different word features, languages, and techniques. The text presents practical examples, including code to run the experiments, generated datasets and validation sets (see <https://github.com/WordsGPT/psycholinguistics_framework>). Importantly, our discussion also includes some dead ends we encountered: configurations that led to useless results. The main goal of this framework is to reduce the effort needed to run experiments on LLM data generation so that a researcher only needs access to run LLMs and a small dataset of a 1000 human estimates for validation. Having those, the effort of running experiments and producing LLM estimates should be comparable to that of a quantitative research analysis in psycholinguistics.

Figure 1 summarizes the five steps of the methodology. In the first step, validation data is collected which will be used to evaluate the quality of the LLM estimates we obtain. The second step involves selecting the LLM, its configuration, and the prompt design. In the third step, the first estimations are obtained from the chosen LLM. In the fourth step, the validation data is divided into a training and test set. The training data is used to fine-tune the LLM; the test set is used to see how much the fine-tuning improved the estimates provided by the model. Finally, in step 5 the fine-tuned model is used to estimate new words for which no human data is available.


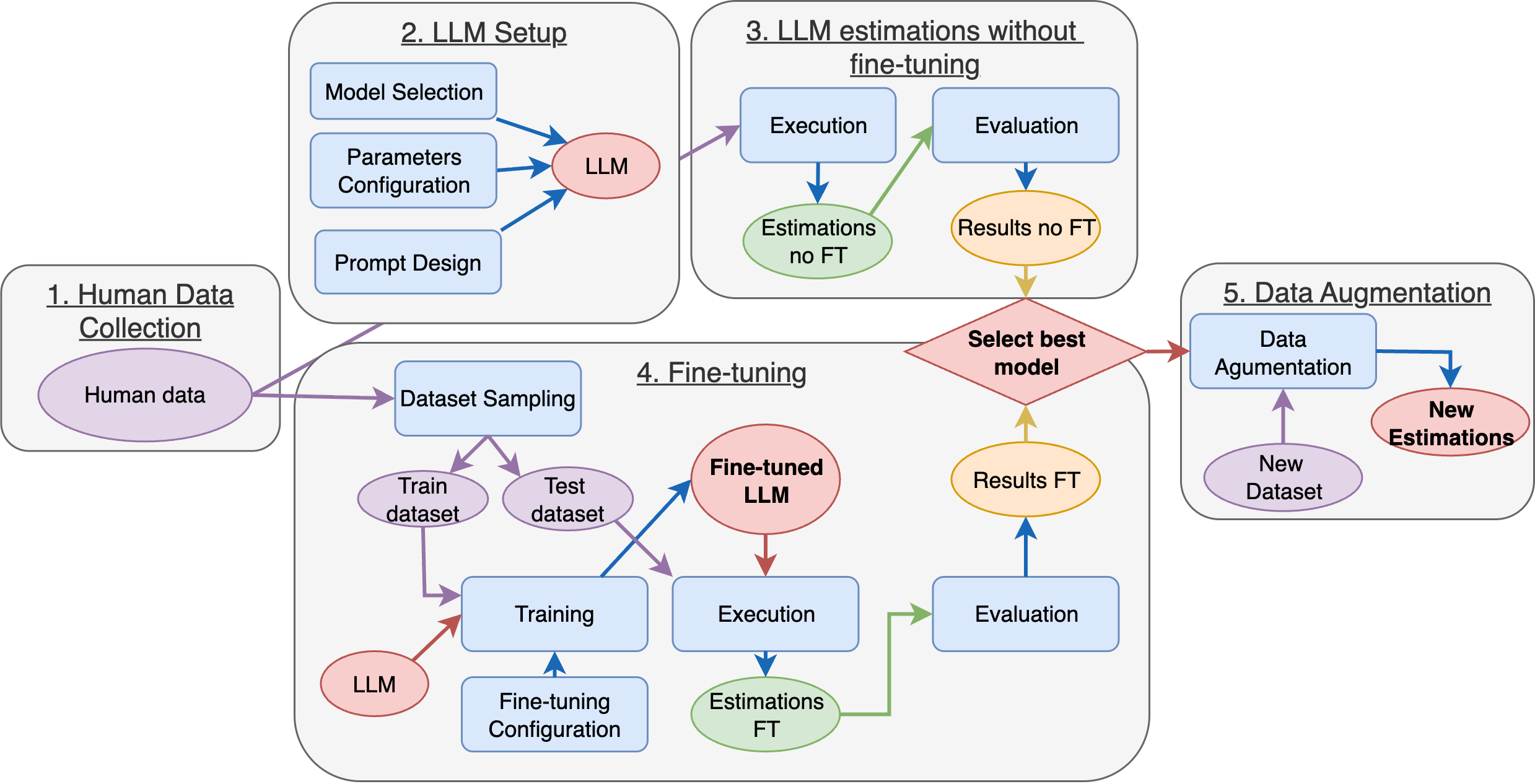


*Figure 1: Estimation of word characteristics with fine-tuned LLMs methodology*

To make this section hands-on, we include a case study on estimating familiarity values in English. We have applied this methodology to other case studies in different languages and with different word characteristics, and we have seen the best results in English.

The advantage of a pipeline protocol is that it reduces the time needed to perform experiments, promotes reproducibility, and reduces the chance of errors, since everything is controlled by configuration files and the execution is automated via scripts. If you want to replicate the results, you must first download the code and install the required Python libraries (step-1), then create a folder for your case study and a “config.yaml” file that will serve as the main configuration file for the experiments (step-2). There is a template of this “config.yaml” file in the code. We have already created the folder “familiarity_english” with all the code and configuration files that can serve as a reference.

- 1. *Collecting validation data*

There is little point in generating LLM estimates of word features, if you do not have data to evaluate them. Computers are extremely good at generating output, but there is little inherent guarantee that the data is of any use for the question you want to address. Even the best documented program with lots of face validity can produce useless output if a small programming error was made in one of the hundreds of lines of code. LLMs can be used to check code and avoid mistakes, but quality control works best if you have a pipeline that always compares the output to what is expected to happen.

The evaluation of predictions is especially important because, although LLMs have proven to be promising tools, their performance varies depending on the type of task. For example, models that seem very powerful in linguistic reasoning can show limitations in seemingly simple tasks like counting letters in words (Conde et al., 2025b). Predicting the performance of an LLM for a specific task is difficult, so having a real and well-annotated criterion dataset becomes an essential requirement to validate its usefulness. If you do not have a validation criterion, you cannot expect people to have faith in the data you produce, and you have no way of optimizing your work.

There are three ways to obtain validation data and we encourage researchers to use all three of them, because this provides them with rich feedback.

- - 1. *Human data in the target language*

The first validation criterion consists of human data. If you claim that the estimates you obtain from an LLM have psycholinguistic value, you must show their usefulness to approximate human data. Such data can be human ratings or other performance indices (e.g., reaction and accuracy in a word processing task). If you are lucky, you can find datasets other people already collected. It is worthwhile to do a deep search for these, as each dataset is a goldmine for you. One dataset is good, but two or more are better, because this allows you not only to look at the correlation of your LLM output to the human data, but also to see how much a set of human data correlates with other human data. A low correlation with a human dataset indicates poor LLM performance, but less so if different datasets also show low correlations: in such cases, it becomes clear that the human dataset is suboptimal or that the construct simply exhibits low agreement among humans.

If no human dataset can be found (or if only one can be found), it is necessary to collect new data. Sometimes this dataset can be rather small if the question is very specific. For instance, Martínez, Molero et al. (2025) collected new human data to verify the quality of LLM estimates of valence and arousal for English words. Because all other evidence pointed to a high quality of the estimates, it was enough for the authors to take 100 words with a wide range of valence estimates and 100 words with a large range of arousal estimates and to present them for a rating study to some 20 people each. Correlations between the LLM estimates and the human ratings were higher than 0.9, suggesting that LLM-generated estimates were indeed high quality.

That said, when possible, it is best to have human data for a few thousand words. Generally, humans can provide trustworthy ratings of word features at a rate of 1000 per hour (based on Conde et al., 2026). So, the (time) investment is not too much of an obstacle. The number of raters you need depends on the correlation between the ratings of the different participants. For Likert ratings, these tend to be high (at least 0.2), meaning that most of the time 10-20 raters will be enough to get reliable ratings. A sensible approach is first to collect data from 12 raters and collect the reliability of these ratings. This can be as simple as Cronbach’s alpha, available in several R packages (e.g., psych; Revelle, 2015) and in almost any statistical package. The reliability should be larger than .8 and if it is not, it is worthwhile checking whether there may be one or two participants who responded carelessly. You can see this by calculating the correlation between the ratings of each participant with the average rating of the remaining participants (again present in nearly all packages that contain Cronbach’s alpha). If a participant responded in line with the other participants, there will be a positive correlation between the participant’s ratings and the ratings of the others. If the participant responded carelessly, there will be no correlation between their ratings and those of the others.

If the reliability is smaller than .8 and there are no signs of careless responding, then there is divergence in the responses of the participants and more raters must be tested before the average scale value has the desired reliability^[[1]](#footnote-1)^. You can estimate how many extra participants you need to test with the Spearman-Brown prophecy formula: n = 0.8*(1-r_obtained_) / r_obtained_*(1-0.8). If your ratings study has a reliability of .6 after 12 participants were tested, you will need 0.8*(1-0.6)/0.6*(1-0.8) = 2.67 as many participants in total or 2.67*12 = 32 participants (thus, 20 extra participants to test).

A reliability of 0.8 means that the maximum correlation you can find between your LLM estimate and the human ratings is $\sqrt{0.8}$ = 0.89. Most of the time it will be lower, just like the correlation of your ratings with those of other researchers is unlikely to be higher than 0.8 (the maximum correlation between two variables with less than perfect reliability equals $\sqrt{r_{1}*r_{2}}$; r_1_ = reliability variable 1, r_2_ = reliability variable 2). If you find this ceiling level too low, you can test extra participants until you have a reliability of 0.9, but this will require (many) more participants.

When collecting human ratings, it is good to keep in mind that data gathering should not make it easy for participants to use AI. The days when participants could be given a spreadsheet and asked to fill in the ratings are over, unless you can fully trust your participants. Even tools that require participants to click the value for each item online may no longer be fully safe, given that apps exist that automatically generate an LLM answer for multiple-choice questions or Likert scales. One way to check for these is to make sure that the data of participants do not correlate more with a zero-shot LLM from the most popular public tools than with the other participants.

For the English familiarity case study, we took existing human data based on the 2,545 words for which there are human familiarity ratings in both the MRC database (Coltheart et al., 1981) and the Glasgow database (Scott et al., 2019). In the step-3) you have to save the dataset in XLSX or CSV format in the “data” folder with at least two columns: the word, and the estimation value.

- - 1. *Translation of data from another language*

A second way to get validation data is by translating rated words from another language. English, Dutch, Spanish and Chinese are languages with large databases of human data (e.g., ratings of age of acquisition, concreteness, valence, arousal, familiarity; or performance-based measures, such as word prevalence and lexical decision responses).

Something that works well is to translate words from the source language (e.g., English) into the target language you are interested in (e.g., German) and then back to the source language (English). Words with the same back translation as the original words are words that have very similar meanings in both languages. Forward and backward translation can be automatically (e.g., with Google translate, DeepL, or an LLM).

- - 1. *Other AI estimates*

Finally, there is a chance that some AI-based dataset already exists for the language you are interested in. This is the case, for instance, for word concreteness, valence and arousal, where researchers have calculated estimates for 50+ languages based on semantic vectors (Buechel et al., 2020; Hollis et al., 2017; Plisiecki & Sobieszek, 2024; Solovyev et al., 2022; Thompson & Lupyan, 2018; Wang & Xu, 2024).

AI-based estimates do not help you determine the efficacy of your own LLM-generated norms in predicting human judgments, but at least they provide you with information about how good your estimates are relative to the existing AI standards. Ideally, you find that the new estimate outperforms the existing measures, thus correlates more with the validation criteria.

- 1. LLM Setup

Once you have validation data, you can proceed to generating estimates with the LLM you have for the language you are interested in. Again, there are many choices and decisions to make.

- - 1. Model selection

Model selection is a crucial and non-trivial task given the wide variety of options currently available. There are models of different sizes, costs, and task orientations, so choosing the most appropriate one for a specific problem requires careful analysis.

To date, the models that have provided the best results for us are the commercial LLMs GPT-4o and GPT-4o-mini, both from OpenAI. Importantly, by “best” results we mean the estimates that provide the highest correlations with human ratings. So, they are limited to their usage as psycholinguistic norms. Researchers interested in *how* models produce specific estimates and which variables influence them may benefit more from the use of an open-source, research-based model, even if the output correlates less with human ratings. Further, as noted above, we expect that the performance of open-source models will continue to improve in the coming years.

It is important to highlight that the most expensive or powerful commercial models do not always offer the best results. For example, we obtained worse results with GPT-4.5 than with GPT-4o and GPT-4o-mini. The alignment of the model with the cognitive and linguistic characteristics of human language processing is a key factor in achieving good results and is not always linked to the price or the size of the model. In the case of GPT-4.5, the model has been focused on improving programming capabilities, a task distant from natural human language processing. In a similar way, using the new reasoning models does not necessarily provide significant advantages since prediction of word features does not seem to require complex reasoning capabilities. Again, this reinforces the need for validation data in making an evidence-based choice of model.

Another relevant decision is the choice between open weights models and commercial models. Open weights models require somewhat more complex technical setup, since they typically need to be downloaded, installed, and managed on private or public infrastructure. Having said that, increasingly graphical user interfaces, such as Anaconda AI Navigator, are becoming available that reduce the technical skills needed by researchers to deploy open-source models locally on their systems. Alternatively, LLMs can be accessed through providers that facilitate access (for both commercial and open weights models), such as the HuggingFace Endpoints Hub^[[2]](#footnote-2)^. In contrast, commercial models usually offer access via API, which simplifies use but introduces some limitations. A significant disadvantage of commercial models is the lack of access to weights and intermediate layers, making it difficult to carry out explainability studies. Additionally, there is a risk of losing access if the company decides to change or even deprecate a given model, which interferes with the reproduction and extension of published results. A recent example of this situation is the discontinuation of GPT-4.5 access. Also, a university or other funding source may decide to stop paying for an outdated version and switch to the new one, whenever it becomes available.

Finally, it is essential to document and keep the exact version of the model used. Model providers often release periodic updates of the same model that alter model behavior. For example, the version “gpt-4o-mini-2024-07-18” indicates the specific variant of GPT-4o-mini deployed on that date.

Model selection is neither simple nor unique. We recommend consulting the literature to identify the most suitable models according to the task’s nature and to perform comparative tests with different models and configurations to choose the optimal option. It is also a good idea to keep a time-stamped copy of each validated estimate you obtain, so that you can keep on using it when the model changes or is no longer available.

A golden rule is to never trust estimates that have not been subjected to the validation checks, even if the new estimates are a rerun of a program that produced estimates that correlated well with human estimates in the past. **Validation should be an inherent part of the pipeline you use whenever you obtain AI estimates of psycholinguistic features.**

Within the English study we selected the commercial model GPT-4o-mini (gpt-4o-mini-2024-07-18, from OpenAI) and the open weights model Llama3.1-8B (from Meta)

- 1. Collecting LLM estimates

Once you have decided which LLM model you are going to work with, you will need to make a number of decisions about how to collect the estimates. Here we mention the most important ones we’ve encountered in our research so far.

- - 1. Parameter configuration

The most important parameter for estimating word features within an LLM is temperature. Other parameters can usually be left at their default values unless a specific problem is detected during testing that justifies adjusting them. There are several strategies for configuring temperature:

1. **Temperature = 0.** Setting temperature to 0 aims to make the process as deterministic as possible, thus facilitating the replicability of the experiment. Still, it is good to know that temperature = 0 does not mean absolute determinism because of the nondeterminism in the commercial models hosted by companies like OpenAI due to the use of finite precision in the arithmetic operations which depend on the execution order (Fu et al., 2026). You can consider this the reliability of the AI estimates. Most of the times, we have found correlations above .96 between two runs. When determining the reliability of the AI estimates, it is important to make sure that the order of items differs between the runs, so that you check to what extent the estimates are influenced by previous estimates made.
2. **Temperature = 0 with logprobs.** This configuration is especially recommended when the task involves predicting an integer that is represented with a single token^[[3]](#footnote-3)^. In these cases, it is possible to obtain the logarithmic probabilities (logprobs) of the top-k tokens and calculate the weighted average to capture the complete distribution of possible responses, not just the most likely estimate. This approach provides more granular estimates. When the required output is non-integer (e.g., numbers with decimal places), it is not advised to use logprobs because the output is likely to consist of multiple tokens, and obtaining the full log-probability tree is extremely computationally intensive, as its complexity grows exponentially.
3. **Temperature ≠ 0**. In this case, the result you obtain will differ between runs, because a temperature different from 0 indicates that the estimation is not deterministic and was generated using the estimated probabilities of the possible output tokens. In other words, the test-retest reliability of the estimates will be reduced (you can easily test this by calculating Cronbach’s alpha of different runs with temperature set at a particular value). When a temperature different from 0 is chosen, you can get more reliable estimates by repeating the experiment a number of times and calculating the average values (just like the average human rating is calculated). Authors may prefer this approach, if they think randomness is an inherent part of estimation and they suspect that the value obtained with temperature = 0 does not correspond to the outcome based on averaging several noisy runs. Again, having validation criteria is a great help here to make informed decisions. A possible strategy is to run the model multiple times with the default temperature and capture statistics such as mean, mode, median, and standard deviation of the predictions obtained as independent samples. This allows capturing the inherent variability of generation with non-zero temperature. If resource limitations prevent multiple runs, it is preferable to use temperature 0 to ensure reproducibility of the experiment.

The framework is configured with Temperature = 0 and registers the logprobs. It uses the logprobs when possible, i.e., when the output is limited to an integer between 0 and 999.

- - 1. Prompt Design

Prompt design consists of providing instructions to the LLM to make the estimation. To some extent, LLMs have made it easy to write prompts, because we can use the same instructions as we give to people. Indeed, a good starting point is to use the instructions used in a classic human rating study (or the rating study you ran to obtain validation data).

At the same time, it is good to keep in mind that small changes in the prompt may affect the quality of the results. For example, model performance may benefit from the inclusion of certain background information in the prompt. Take the following simple prompt:

1) *Rate the familiarity with {Word} on a scale from 1 to 7. Return a number.*

This prompt supposes that the model attaches the same meaning to the word familiarity as you do. It also supposes that the model shares your concept of a 7-point scale and how it should be used to extract the maximum of information, given the range that can be expected. For instance, if you only intend to present words likely to be known by people, a good interpretation of answer 1 is “a word I may have come across before but I do not know what it means”. Then the entire range of 1 – 7 is used. In contrast, if the stimulus list includes word unlikely to be known by people, the meaning of answer alternative 1 should shift to “a word I’ve never seen or heard before”, in order to include the entire range of values that will be encountered. Also, what is the proficiency level you have in mind: that of an adult native speaker, a child, or a second language learner?

It is a good idea to make the above assumptions explicit by expanding the prompt (as is done in most human rating studies as well). For instance, the following prompt may be better:

2) *Complete the following task as a native speaker of English. Familiarity is a measure of how familiar something is. An English word is very FAMILIAR if you see/hear it often and it is easily recognisable. In contrast, an English word is very UNFAMILIAR if you rarely see/hear it and it is relatively unrecognisable. Please indicate how familiar you think this English word is on a scale from 1 (VERY UNFAMILIAR) to 7 (VERY FAMILIAR), with the midpoint representing moderate familiarity. The English word is: “{Word}”. Only answer a number from 1 to 7. Please limit your answer to numbers.*

In this example (based on Scott et al., 2019), we are priming the LLM to yield the information we have in mind (remember that the LLM produces the most likely token given the preceding context). We can investigate the effect of the more detailed prompt by obtaining estimates with both prompts. If prompt 2 is better than prompt 1, it will provide estimates that correlate more with the validation dataset(s). If there is no difference between the prompts, we can limit ourselves to the shortest prompt to reduce processing time and overall cost. If prompt 2 is better than prompt 1, we can ask ourselves whether other information may further improve it. For example, would the prompt become better if we add a few examples of words that are expected to get an estimate of 1 and words that are expected to get an estimate of 7? If providing that information does not improve performance (much), it may be better to leave it out, as you are interfering less with the model’s training regime. On the other hand, if the extra examples increase the correlations by a nontrivial amount (e.g., +0.05) you may want to include them in the prompt, as it brings the estimates closer to human evaluations.

In addition to *content*, the *format* of the prompt also matters. Some examples of formatting choices include:

1. Asking the model to return both the word and the number (instead of just the number), so that you protect yourself against alignment issues in case the LLM does not provide a value for a few words in the list (which we often see in our experiments).
2. Asking the model to return a JSON with a specific format, including the word: *The output format must be a JSON object. For example:* *{"Word": "{Word}", "Familiarity": // Familiarity of the word expressed as a number from 1 to 7}* This command helps to manipulate the results later through coded programs (i.e., scripts).
3. Including the word in the prompt without a colon, without quotation marks, and in the middle of the sentence: *[…] Please indicate how familiar you think the English {Word} is on a scale from 1 (VERY UNFAMILIAR) to 7 (VERY FAMILIAR), with the midpoint representing moderate familiarity][…]*
4. Making the prompt in a different language. Unlikely to lead to better results if you want estimates for the English language, but something you may want to try out if you are looking for estimates in a language that did not figure prominently in the training and fine-tuning of the LLM. Sometimes you may get better estimates with English instructions than with instructions in the language you are investigating.
5. Asking for decimal numbers: *Only answer a number from 1 to 7. Please limit your answer to numbers, it may include up to two decimal places.* It is recommended to limit the number of decimal places, as otherwise the model might hallucinate and generate out of range numbers.
6. Asking for an integer from a larger range, e.g. from 10 to 70, so that we can divide the estimate by 10. *[…]Please indicate how familiar you think each English word is on a scale from 10 (VERY UNFAMILIAR) to 70 (VERY FAMILIAR), with the midpoint representing moderate familiarity. […] Only answer a number from 10 to 70. Please limit your answer to numbers.*
7. Adding a few examples to the prompt (few-shot). Including examples with the expected results in each prompt can help the LLM understand the task better. It is important to provide representative examples that cover all possible values. In the tutorial we proved this technique with three examples *Complete the following task as a native speaker of English. Familiarity is a measure of how familiar something is. An English word is very FAMILIAR if you see/hear it often and it is easily recognisable. In contrast, an English word is very UNFAMILIAR if you rarely see/hear it and it is relatively unrecognisable. Please indicate how familiar you think this English word is on a scale from 1 (VERY UNFAMILIAR) to 7 (VERY FAMILIAR), with the midpoint representing moderate familiarity.* *For example, "imam" has a familiarity of 2, "theology" has a familiarity of 4, and "fridge" has a familiarity of 7. The English word is: "{Word}" […].*

These are but a few prompts we tried out and the strategies we found useful. The number of possibilities is much larger and possibly unlimited. In addition, a prompt may work well for one task and badly for another, or may perform differently across LLMs.

In step-4) include all these prompts in TXT files stored in the folder “prompts” with one file per prompt. These files will serve as templates for building all the queries to the LLM. You have to use the *{Word}* expression in the parts where the specific word to estimate would be replaced.

Estimates for a new variable or a new language may benefit from experimentation with multiple options to see what effects they have on the outcome. Of course, this introduces the risk of overfitting (or “prompt” hacking); at the same time, optimal engineering often requires some degree of experimentation (Ward, 1998). Researchers can ameliorate this risk by preserving some amount of “held-out data”, which is not used during this experimentation phase—much like a typical “test” set in machine learning. Alternatively, or additionally, researchers could collect entirely new data to validate the estimates.

With well-established languages and variables, a straightforward approach is simply to use the instructions given to humans, i.e., those used in the published article. At the same time, the instructions that are most helpful for humans may not be the instructions that produce the LLM-generated judgments that most strongly correlate with human judgments; thus, some experimentation can be helpful, depending on the researcher’s goal.

Future research is likely to limit the search space of effective prompts. So, it is good practice to review the state of the art in search of the best prompts, to be transparent about the strategies you tried out, and to run validation tests when you think you’ve achieved a major break-through.

- - 1. Execution

As we outlined before, it is not a good idea to work with an interactive web interface to obtain LLM estimates of word features, unless the interface used by the researcher allows settings adjustments to variables such as temperature, top P values, and the continuity of past messages.^[[4]](#footnote-4)^ The most user-friendly web interfaces have been developed to make interacting with an LLM intuitive and pleasant, so they do not give you control over the parameters, and the estimates are likely to be affected by what you did before because they shaped the context window within which you are working. This phenomenon, known as context contamination, can introduce unwanted bias in the results, affecting the validity of the generated estimates.

It is much better to use the API. By using the API, each query is sent as an independent call, where the model always starts “from scratch” without memory of prior inputs. This approach ensures that each prediction is unconditioned by previous estimates, which is key to guaranteeing consistency and reproducibility of the experiment. The use of the API in commercial models is not free; it is charged per input and output token. You must register and obtain an API key. There is a wide range of prices among models, and access to LLMs varies internationally, as do costs. Nonetheless, for most western-based university researchers, today GPT-4o-mini costs $0.15 per million input tokens and $0.60 per million output tokens, while GPT-4o costs $2.50 (input) and $10 (output) per million tokens. One million tokens is equivalent to about 750,000 words in English, including both the input prompt and the model’s response. Any other language different from English, will be more expensive as the models represent words with more tokens^[[5]](#footnote-5)^. There are online calculators that estimate the cost of running experiments^[[6]](#footnote-6)^. For instance, running prompt (2) on 100,000 words would cost $2.19 with GPT-4o-mini and $35 with GPT-4o in English. The price is not always aligned with quality and depends on the provider. From our experience, GPT-4o-mini is sufficient for linguistics tasks, certainly if fine-tuning is used.

LLM providers offer different interaction modes through their API. The most direct method is synchronous processing, where an individual request is sent and the response is received immediately. This option is useful for small tasks, quick validation, or cases where interactive inspection or debugging of results is needed. However, when working with large volumes of data, it is more efficient to use the asynchronous mode, known as batch processing. In this mode, the user submits a file with all the questions to be processed, and the provider returns the responses within a maximum of hours (typically 24). This approach does not require keeping local infrastructure running and it is usually cheaper.

Despite its advantages, batch processing requires some consideration. Some entries may fail during the process, so it is essential to verify that all requested estimates have been generated. Additionally, it is important to check that all responses have the correct format, since occasionally the model may deviate from the instructions, producing unexpected outputs or structural errors. For example, it has been observed that when asked in the prompt to also return the word, the model changes the input word to another word in the output. In such cases, it is recommended to rerun the failed entries, either by making new individual calls or generating a new batch exclusively with those examples.

The framework is prepared to run in batch mode using the API. In step-5) configure the “apis.env” with your API key (you have a template of this file in “apis_example.env”). If you are going to share the code from your experiments, make sure not to upload your API_key, as someone else could use it in your name.

Then in step-6) you have to configure the config.yaml, including the name of the experiment, the XLSX with your dataset, the name of the column where the words to estimate are, the file with the prompt to evaluate, and the LLM to use. As an example, for the first prompt the content would be:

*experiments:*

*familiarity_english_v01_short_prompt:*

*dataset_path: "Glasgow_MRC_joint_norms_inner_join_english.xlsx"*

*dataset_column: "Word"*

*prompt_path: "english_v01_short_prompt.txt"*

*model_name: "gpt-4o-mini-2024-07-18"*

In step-7) run the program “python3 prepare_experiment.py <EXPERIMENT_PATH> <EXPERIMENT_NAME>” replacing EXPERIMENT_PATH with the name of the case study folder (“familiarity_english”), and EXPERIMENT_NAME with the name of the experiment (“familiarity_english_v01_short_prompt”). As a result, a file with all the queries to be made to the model will be generated in a folder called “batches”. Check in this file that the generated prompt is what you expect. If your dataset contains more than 50,000 words, several files will be generated due to OpenAI API limitations.

Then (step-8) run “python3 execute_experiment.py <EXPERIMENT_PATH> <EXPERIMENT_NAME>” to send your petitions to the model’s API. In batch mode, it may take up to 24 hours to complete the results, meanwhile you can turn off your computer as nothing runs on it. To download the results, you have to access the OpenAI website – batches section.

In step-9) download the results and save them in a folder called “results.” The file with the results contains a lot of information related to the model execution. To make the results manageable, run “python3 generateResults.py,” which will process all the results and save them in a new XLSX file for future analysis.

- - 1. Evaluation

Evaluation consists of assessing how accurate the estimates are compared to the validation measures. We recommend calculating both Spearman and Pearson correlations (Conde et al., 2025a). Most of the time they will not differ much, in which case you know the correlations can be relied on. Sometimes, however, the correlations will differ considerably. This is most likely when the distribution of a variable is asymmetric, for example when you have a variable with many low values and a few high values (e.g., how much is each word related to smell?). To understand differences between Pearson and Spearman variables, it is good to know that the Pearson correlation gives more weight to extreme values (i.e., those at the low and the high end), whereas the Spearman correlation gives more weight to (possibly small) differences around the mode. A higher Pearson correlation than Spearman correlation indicates that the correlation largely depends on words at the high and/or low end of the scale. A higher Spearman correlation than Pearson correlations indicates that the correlation is mainly situated in the middle of the scale with deviating items at the extremes.

To make things more tangible, Figure 2 presents some results for the prompts we discussed with respect to English familiarity. The data are based on the 2545 words for which there are human familiarity ratings both in the MRC database (Coltheart et al., 1981) and Glasgow database (Scott et al., 2019). The Pearson correlation between these two datasets is r = .79; the Spearman correlation is ρ = .80. These are the baselines to compare the LLM estimates with. GPT estimates show similar results in its best configurations, with Spearman correlations of ρ = .79 (MRC) and ρ = .82 (Glasgow), and Pearson correlations of r = .76 (MRC) and r = .81 (Glasgow). As mentioned before, the way we ask the model is important. For example, the short prompt without explanation (prompt-v01) produced the worst estimations. In fact, for many words, it provided additional text before giving the estimate. In the analysis, we did not observe a difference between a prompt asking the model to return both the number and the word versus only the number. Marking the word in the prompt is relevant, since without a colon and quotation marks, the Spearman correlation dropped by .12. Rescaling strategies had a negative impact here, and writing the prompt in Spanish did not show a negative effect. What induced a significant improvement, up to .10 in correlation, was using logprobs. The best prompt when considering both datasets was the few-shot one. We included three reference words: “imam” (familiarity of 2), “theology” (4), and “fridge” (7). It is important to note that these results are not the ground truth. In other scenarios, we have obtained very different outcomes, and what works here may not work in other cases. With Llama3.1-8B (the open-weights model) we obtained worse results. In fact, there are large differences between the different strategies tested. The one that worked best was the standard prompt with logprobs, but it was .20 points below its counterpart in GPT-4o-mini. It is also noteworthy that the use of logprobs was also very effective in Llama, since the correlation without logprobs in Llama3.1-8B was .30 points lower.


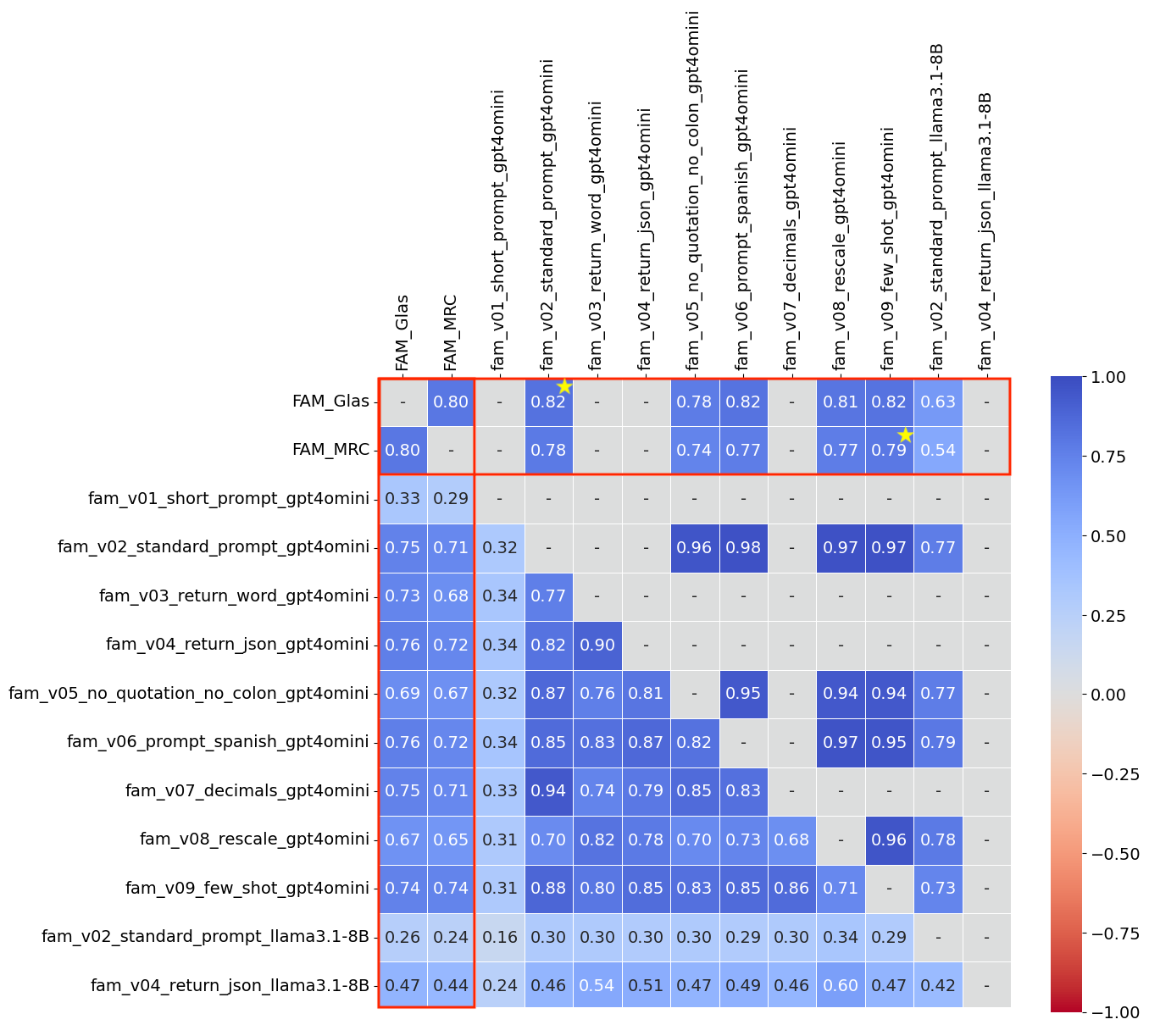


Figure 2: Spearman correlations of the GPT and Llama prompts with Glasgow and MRC English familiarity databases (over the 2545 common words). Above the diagonal: correlations calculated with logprobs (when possible); below the diagonal: correlation using the output of the model.

Correlations mainly reflect rank-order similarity and do not necessarily guarantee agreement on the absolute scale of the ratings. To complement correlation-based analyses, it is recommended to report additional metrics of absolute agreement between model predictions and human ratings. Specifically, we compute the mean absolute error (MAE), which quantifies the average magnitude of prediction errors regardless of direction, and the standard deviation (SD) of the errors, which reflects their variability across items. In addition, we recommend examining the mean signed error (MSE) to assess whether the model shows a systematic tendency to overestimate or underestimate values, as well as the distribution of errors across the scale to determine whether prediction errors are uniform across different value ranges. For instance, for the best configurations we obtained for MRC an MAE of 1.12, SD of 0.51 and an MSE of +1.10, indicating a slight tendency to overestimate familiarity values. In the case of Glasgow, we obtained similar results with an MAE of 0.87, SD of 0.53, and MSE of +0.86. Regarding the effect across the distribution, we observe that LLMs tend to produce more 5–7 ratings (Figure 3).


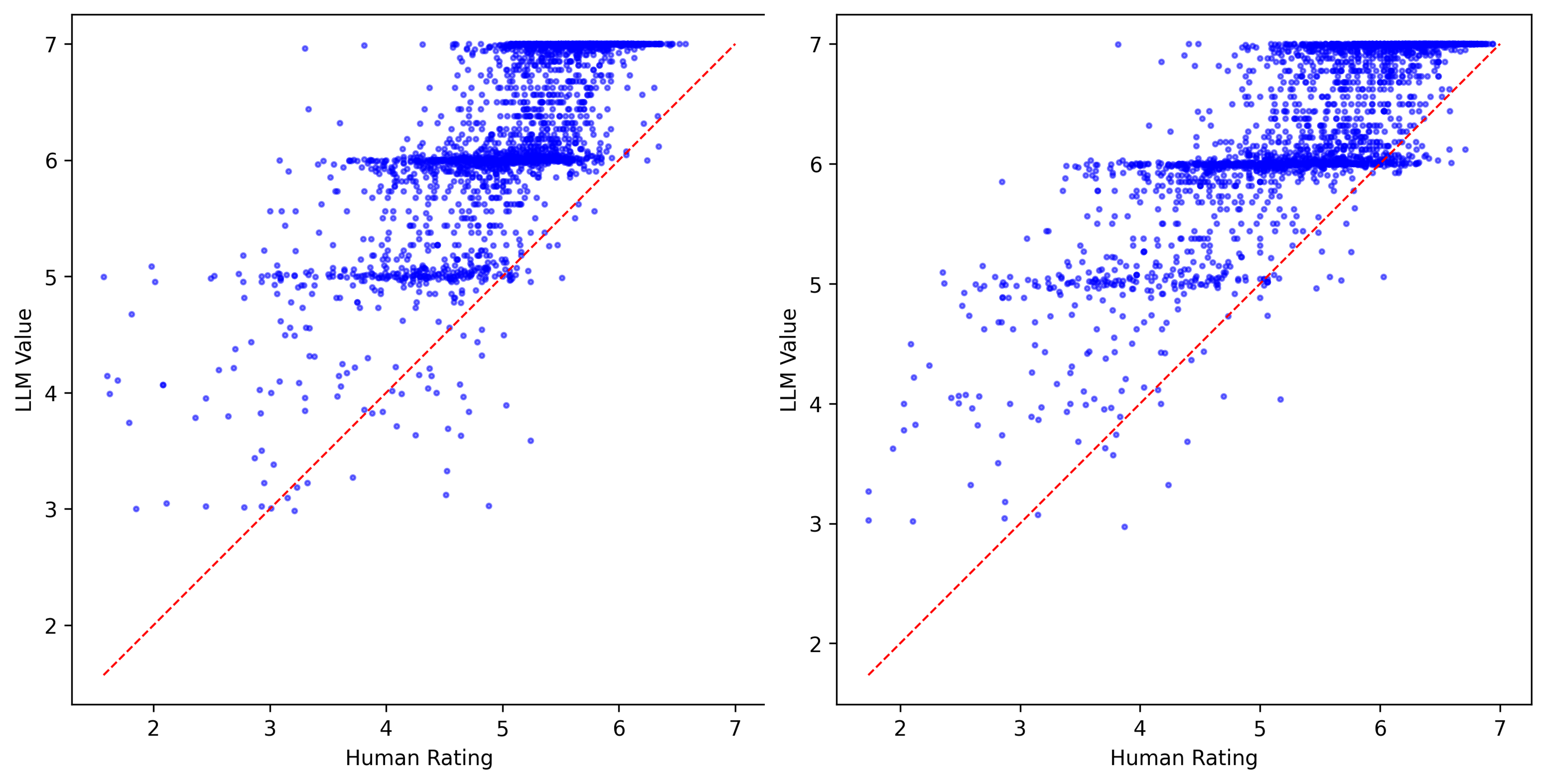


Figure 3: Scatter plot of human ratings vs LLM estimations for MRC vs prompt_v09-with_logprobs (left) and Glagow vs prompt_v02-with_logprobs (right).

- 1. Fine-tuning the model

Depending on the quality of the LLM estimates, you may look into improving them by fine-tuning the model. In fine-tuning, the model receives feedback about the expected values, so that the weights can be adjusted and the output more closely resembles the desired one. Such fine-tuning is not needed if the model already produces estimates on par with what you observe in humans (as in Figure 2), but is worth considering when the estimates from step 3 are below expectation. If is not a fail-safe solution, but we found that the effect sometimes can be quite pleasing.

- - 1. Dataset sampling

Fine-tuning requires data to feed to the model. Most of the time, the data will be a part of the validation data you collected in step 1. An alternative strategy is to collect new human data based on the results of step 3. For instance, we could extract a sample of interesting words based on the familiarity estimates obtained in Figure 2. These can be words that had different ratings in MRC and Glasgow, words for which the different prompts gave divergent estimates, words with a big difference between the familiarity estimate and word frequency. These are interesting words to collect human data for, in order to bring the model more in line with human opinion.

At the same time, it is good to keep in mind that it is not a good idea to feed the model only with exceptional cases, as this risks distorting the estimates for the rest of the words. A good strategy is to combine some interesting new words with words for which the model already gives good output.

The number of words that are needed for fine-tuning depends on the noise in the data. For reaction times (which are inherently noisy) you will need more feedback than for ratings on a Likert scale. We found that not much gain is made after feedback on 2000 words for Likert ratings of AoA (Sendín et al., 2025), whereas 5000 words were needed for fine-tuning a model to predict reaction times in a lexical decision task (Martínez et al., 2025).

The more data you have to fine-tune the model, the more closely the model’s output will resemble the validation criterion. LLMs already have a lot of linguistic knowledge based on their massive pretraining and all that is needed is to bring the output better aligned with that knowledge. So, even if you only have a small number of data to feed back to the model, it can still be worthwhile to test what effect it has.

Importantly, to evaluate the effect of fine-tuning you need a dataset that was not used in the tuning itself. There is little point looking at whether fine-tuning improved the estimates for the words used as feedback, as this will always be spectacular (the model learns what it should return for these particular words). The big question is how much better fine-tuning makes the estimates for *new* words, a process called cross-validation. Cross-validation requires that you collect data for new words after the fine-tuning, or that you split your available dataset into at least two subsets: one for training and another for evaluation. In classical machine learning problems, a standard split of 80% for training and 20% for evaluation is adopted. However, in the context of fine-tuning, this ratio can be more flexible and it may be better to split 60-40, so that you have enough data to test the outcome of the fine-tuning. If you only have data for 1000 words, it is not really reassuring if your quality test is based on 200 words only. In general, we recommend aiming for data for at least 3000 words, 1800 of which can be used for training and 1200 for testing. With sample sizes of more than 1000, the results are unlikely to differ much between different random splits of the data into a training and testing set. In case of doubt, it is always possible to fine-tune a model more than once and look at the extent to which the results depend on the specific training and test samples extracted.

The quality, diversity, and coverage of the dataset are important variables to consider, in addition to the size. Regarding sampling strategies, the most straightforward approach is to simply take a random selection of all stimuli. This works well if each stimulus is equally interesting, but will reduce the information extracted if only a subset of words is of interest. For example, you could take a random sample of a list of 120,000 English lemmas for familiarity estimates. However, given that more than half of these words are unlikely to be known to your participants, you are throwing away much information, because all these words end up in the lowest bin. It may be more interesting, to limit the words to words that are likely to be known to some people, for example based on their frequency or on previous word prevalence information.

Stimulus selection for finetuning also has a dark side, because you are excluding potentially interesting information. For instance, Sendín et al. (2025) finetuned a Spanish model to 3000 AoA ratings obtained by Alonso et al. (2015). These ratings were limited in two ways. First, the words had been selected such that most words were expected to be acquired in the first 12 years of life. Second, participants were asked to categorize all words learned after the age of 11 years to a single category of 11+. This meant that the fine-tuned model could only provide information for early acquired words. All late acquired words got an estimate of 10-11 years old, in line with the fine-tuning regime the model was given.

It is therefore important to always be aware of possible biases and limitations in the training set and to test the fine-tuned estimates on a validation sample of stimuli. With regard to cross-validation, it is important to keep in mind that the value of cross-validation decreases with each additional test you perform. A mistake researchers often make is to try out dozens of cross-validation analyses and keep the best one. Cross-validation involves applying your *chosen* analysis path to a *new* dataset to see how well it generalizes. That is why it is a good idea to always keep one data sample until the very end of your pipeline (or collect new data at that point).

Fine-tuning can also have unexpected positive outcomes. For instance, in experiments estimating word familiarity in German, we observed that fine-tuning did not change much for words typically used in psychological studies, but we observed that the fine-tuned model was much more sensitive to spelling errors in the words (something that often happens in corpus analysis). Whereas the model before finetuning tended to give rather high estimates to recognizable words with small spelling errors, it gave low estimates after having received feedback on 2000 correctly spelled German words (this included the use of capitals for nouns, something typical for German).

- - 1. Fine-tuning prompts

Because feedback is given to the model after each estimate, the prompt used for fine-tuning can be shorter than the one used for an untuned model. There is no need anymore to include the scale end or to give examples of extreme words. Even the definition of the variable (“familiarity” in our example) can be dropped, because the model gradually learns what you want on the basis of the feedback.

Other decisions remain, such as whether you ask for both the word and the estimate, whether you use logprobs or increase the range of the scale, and so on. For instance, it is not clear what the consequences are if you give feedback about ratings with decimal places. One of the consequences we saw is that this seems to reduce the granularity of the estimates relative to the untuned estimates based on logprobs. The model seemed to restrict the number of outputs it gave. More work is needed here.

Finally, the fine-tuning parameters (e.g., batch size, number of epochs, learning rate) must be selected. Platforms like OpenAI provide default settings that are adapted automatically to the training dataset. In our research, we usually use these default settings, but it is crucial to report the parameters that were ultimately chosen to ensure the experiment’s reproducibility.

- - 1. Training and evaluation

A model is fine-tuned by first giving the prompt, asking for an estimate. Afterwards, the model is informed about the output that was expected. This is done by providing the correct output as a target, allowing the model to adjust its internal parameters through backpropagation (the standard method for training neural networks), minimizing the error between its prediction and the desired result.

Once the model has seen all the training stimuli, it must be saved, and the fine-tuned model can then be used to provide estimates for the test stimuli. Evaluation of the fine-tuned model is done by looking at how much more the estimates of the fine-tuned model correlate with the validation criteria for the test stimuli. You can also look at the effect for the trained stimuli, but this effect will always be rather large and says nothing about the quality of estimates for new stimuli.

In case of doubt, it is always a good idea to collect some new human validation data. Two advantages of this strategy are that the new findings cannot be contaminated by information already available on the internet (and possibly included in the training of the model) and that you can limit the stimuli to those that are most informative to test the difference between the untuned and the fine-tuned model (e.g., words for which the estimates differ a lot).

The final evaluation must be carried out using a dataset that has not been used in previous stages of training. This evaluation dataset must be kept completely independent to ensure an objective and unbiased assessment.

Following the English familiarity case study, we decided to split the 2545-item dataset into a training set for fine-tuning (1500 entries) and a test set for validation (1045 entries). The sampling strategy was a random split. We tested three different estimations for fine-tuning: based on the Glasgow norms, the MRCnorms , and the mean of the Glasgow and MRC norms. This resulted in three different models: ft_v01_glas, ft_v01_mrc, and ft_v01_glas_mrc_mean, respectively. The fine-tuning instruction used was v02_standard_prompt.

To reproduce the fine-tunings with the framework, you have to complete the following steps:

First, (step-10) add your fine-tuning to the “config.yml”. Specify the input file with the inputs and reference estimates, the percentage of inputs you want to use for training normalized to one (if the input dataset contains only the training data, set this to 1), the name of column where the reference value is located, the prompt to use for fine-tuning, the base model to be fine-tuned, and the name of the new model. For example, for fine-tuning with the Glasgow estimates, use the following configuration:

*finetuning:*

*familiarity_english_ft_v01_glas:*

*ft_dataset_path: "1_train_random_MRC_Glas.xlsx"*

*train_split_percentage: 1*

*random_state: 42*

*answer_column: "FAM_Glas"*

*prompt_path: "english_v02_standard_prompt.txt"*

*dataset_finetune_name: "Glasgow_MRC_joint_norms_inner_join_english_ft_v01_glas.jsonl"*

*model_name: "gpt-4o-mini-2024-07-18"*

*especial_suffix: "familiarity_english_ft_v01"*

After that (step-11) execute the program “python3 create_finetuning_dataset.py <EXPERIMENT_PATH> <EXPERIMENT_NAME>” to build the file with all the estimates, and in step-12) run “python3 execute_finetuning_dataset.py <EXPERIMENT_PATH> <EXPERIMENT_NAME>” to train the new model. Again, before executing the training it is good practice to check that the file with the fine-tuning data is correct. The framework is programmed with the default OpenAI fine-tuning settings. As before, the fine-tuning does not run on your computer, so you can turn it off. Fine-tuning may take several hours, and once finished, you can access the OpenAI website – fine-tuning section to obtain the model and the final training parameters. The new model is saved on OpenAI’s servers and can be used by referencing it.

Finally, step-13) consists of running the evaluation dataset with the fine-tuned model, following the same steps described in step-6 to step-9.

Figure 4 shows the results obtained when fine-tuning the GPT and Llama Model. In the case of GPT, the best results for each database occur when the model is fine-tuned using its own estimates, reaching a Spearman correlation improvement of 0.10 (Glasgow) and 0.09 (MRC), as we can expect. Additionally, fine-tuning with the other database had a small improvement of 0.03 (Glasgow) and 0.04 (MRC). The best balance was achieved when using the mean of the Glasgow and MRC norms, which yielded improvements of 0.09 (Glasgow) and 0.08 (MRC), making it the best-performing model. This model achieves a higher correlation than the one observed between Glasgow and MRC. The fine-tuned model can be understood as an intermediate point between both datasets. In the case of the Llama model, we obtained a substantial improvement on Pearson correlation of 0.22 on Glasgow and 0.30 on MRC, achieving performance that is similar to, though slightly worse than, the GPT models. This indicates that fine-tuning helps improve estimations in models that perform poorly in their base version.


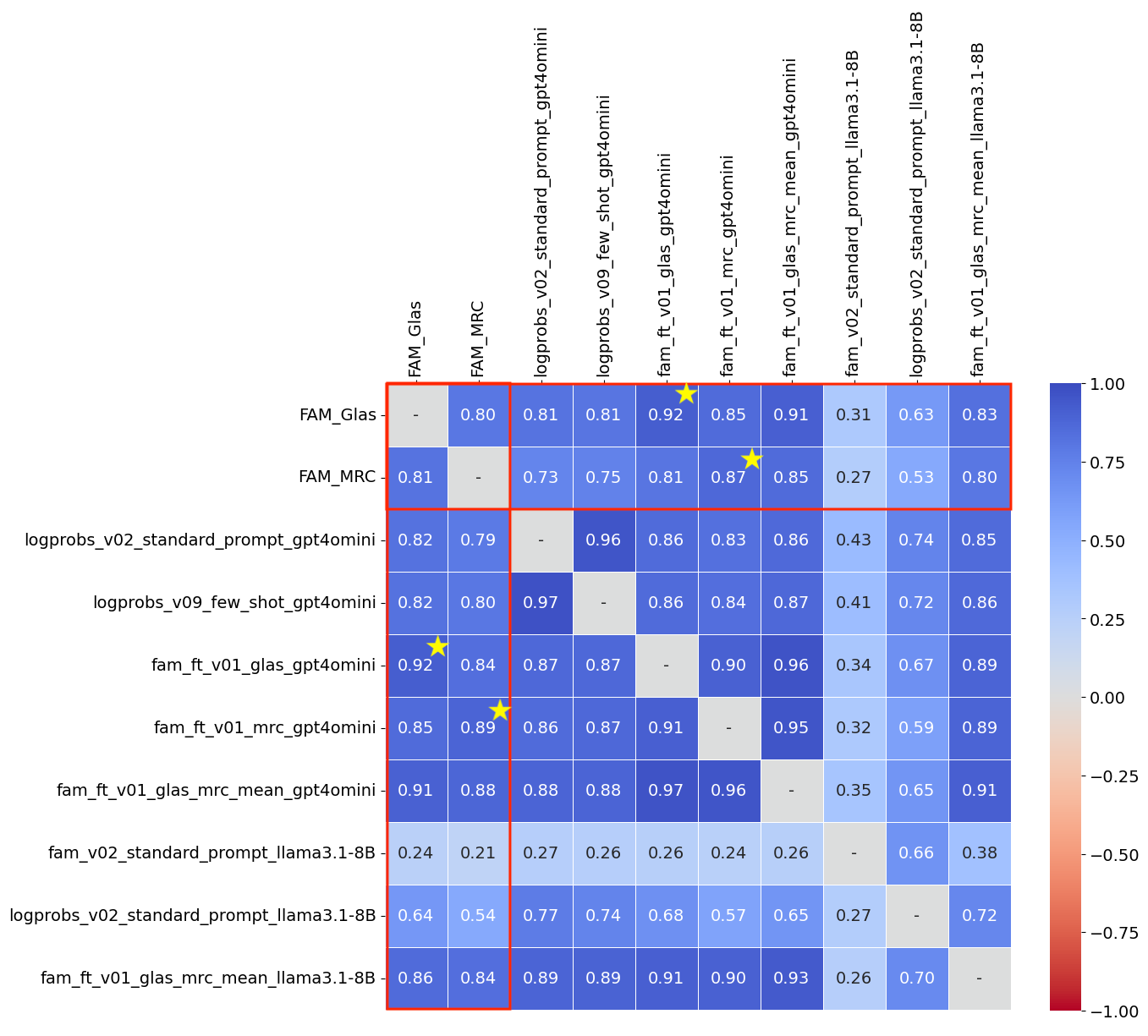


Figure 4: Correlations of the GPT and Llama fine-tuned models with Glasgow and MRC English familiarity databases, v02_logprobs and v09_logprobs prompt versions without fine-tuning, over the 1045 validation words. Above the diagonal: Pearson correlations; below the diagonal: Spearman correlation.

In this case, after fine-tuning, the observed alignment on the test dataset is not limited to an increase in correlation; the MAE, SD, and MSE also decrease, and the analysis of the full distribution shows that errors no longer exhibit a bias toward overrating (Figure 5). Specifically, the best configuration for MRC (ft_v01_mrc) has an MAE of 0.25, an SD of 0.35, and an MSE of -0.01. For Glasgow, the best model (ft_v01_glas) has an MAE of 0.28, an SD of 0.36, and an MSE of +0.00.


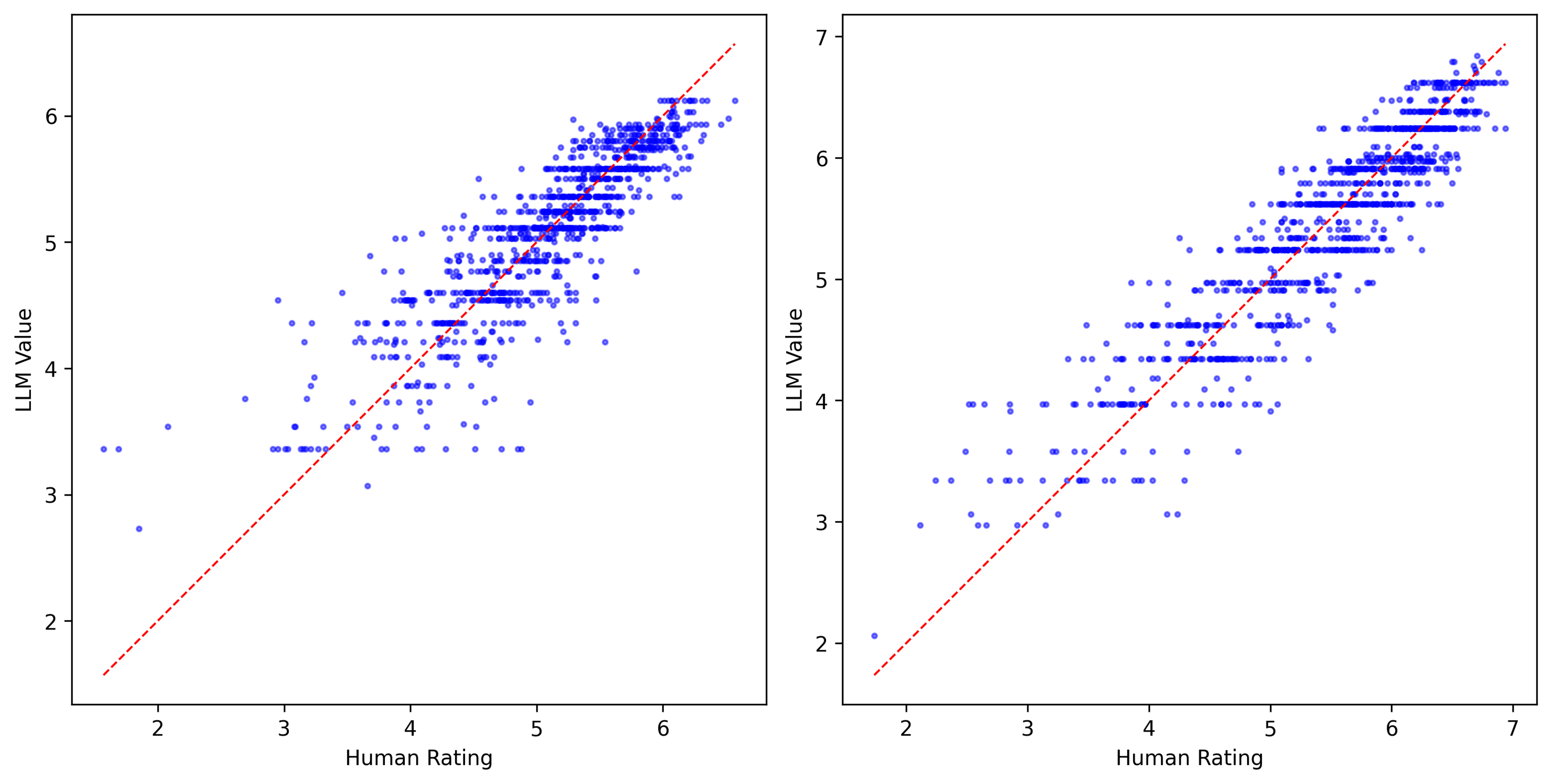


Figure 5: Scatter plot of human ratings vs LLM estimations for MRC vs ft_v01_mrc (left) and Glagow vs ft_v01_glas (right).

- 1. Data Augmentation

Once you are happy with the outcome of the (fine-tuned) LLM you are using, you can extend the estimates to all words of interest. In English, this can be as many as 150,000 words; in languages with many inflected or derived forms and compound nouns written as single words, this number can be larger than 2 million (and potentially reaching a few 100 million possible word forms), as any corpus analysis in German, Czech or Korean will attest.

The procedures for augmenting a dataset are the same as those used in the initial generation of estimates and in fine-tuning. It is good practice to maintain rigorous control over all steps carried out and to document everything to ensure reproducibility. This includes recording the versions of the model used, the prompts employed, the parameter configurations, as well as any pre- or post-processing applied to the generated data.

It is also important to remain aware (and to inform users) about limitations in the augmented data. The main limitation will be that you cannot be sure about the quality of the estimates for word structures not present in the fine-tuning dataset. Above we saw the example of age of acquisition, where you cannot be sure about the estimates for words acquired after a certain age. Another example could be when your model was fine-tuned with lemmas only. In that case you have no guarantee that the estimates are also valid for inflected or derived forms. The same is true for compound words. If they were not present in the fine-tuning you cannot be sure of the quality of the estimates for these words.

The advice is always the same: In case of doubt, collect some new human validation data. This data can be gathered in a very focused way (thus need not be too onerous to collect) and in addition can later be used to further improve the fine-tuning of the model. Indeed, the real contribution of AI to psycholinguistics is not that we replace research on human processing with research on AI processing, but that we leverage LLMs to improve our research on human language processing.

It is also a good idea to keep a time-stamped, well-documented version of each validated word list. If this list contains estimates from a fine-tuned model, it is a good idea to also include the estimates before the fine-tuning. In that way, any potential user can verify what the effect of the fine-tuning was.

To create new data just run again steps 6-9 with the best model and configuration.

## Cross linguistics experiments

As part of the experiments, we asked ourselves whether fine-tuning in one language would improve performance in another language. This would be useful in scenarios where a large database exists in one language but not in another, and collecting real measures is not possible. In this case study, we tested this idea with Age of Acquisition in Spanish and English.

To do so, we created two datasets, one for Spanish and one for English, that were comparable to each other for the experiments. First we aligned Age of Acquisition estimates using Age of Exposure (AoE) using the approach of Botarleanu et al., (2024). For Spanish, data from Alonso et al. (2015, 2016) and Sendín et al. (2025) were merged, cleaned, and adjusted to account for limitations. For example, the Alonso 2015 study has lower estimates and is limited to age 11. Only words that appeared in both MultiLex and AoE, and had a recognition accuracy greater than .98 in SPALEX, were selected. The resulting dataset contained 10,285 high-quality words with adjusted AoA values. For English, the AoA norms from Kuperman et al. (2012) were filtered using the same criteria (present in AoE and MultiLex data, and accuracy in the ECP lexical decision task > .98), yielding 13,094 words. Both datasets were then aligned through linear regression, making them suitable for cross-linguistic comparison.

We randomly sampled 3,000 words from each dataset and fine-tuned two models: one with the English words and the other with the Spanish words. We evaluated three scenarios for each language:

1. Obtain estimations using the base model (GPT-4o-mini)
2. Obtain estimations using the fine-tuned model for the same language
3. Obtain estimations using the fine-tuned model from the other language

The prompts used were as follows:

1. Spanish: La edad de adquisición (AoA) de una palabra se refiere a la edad a la que se aprendió una palabra por primera vez. En concreto, cuándo una persona habría entendido por primera vez esa palabra si alguien la hubiera utilizado delante de ella, incluso cuando aún no la hubiera dicho, leído o escrito. Estima la edad media de adquisición (AoA) de la palabra "{Word}" para un hablante nativo de español. El formato de salida debe ser un objeto JSON. Por ejemplo: {Word: {Word}, AoA: //AoA de la palabra expresado en años, puede incluir decimales}
2. English: The age of acquisition (AoA) of a word refers to the age at which a word was first learned. Specifically, when a person would have understood that word for the first time if someone had used it in front of them, even if they had not yet spoken, read, or written it. Estimate the average age of acquisition (AoA) of the word "{Word}" for a native English speaker. The output format must be a JSON object. For example: {Word: {Word}, AoA: //AoA of the word expressed in years, can have two decimal places}

The same instructions were used in the fine-tunings. As in previous case studies, fine-tuning improved results by around 0.1 compared to estimations without fine-tuning (Table 1). This occurred for both English and Spanish. When using the fine-tuned model from the other language, there was also a positive impact, though roughly half as large (Table 1). It seems that for fine-tuning, language is important but not decisive, as the model can still learn the task in another language, although to a lesser degree. It is also worth noting that English estimations were better than Spanish ones in all three scenarios. We have observed this pattern in numerous experiments, where estimations are better in languages for which the base model has been trained on more data.

|  | GPT-4o-mini | Fine-tuning same language | Fine-tuning other language |
| --- | --- | --- | --- |
| English | .82 | .91 | .86 |
| Spanish | .77 | .85 | .82 |

Table 1: Spearman correlations between human familiarity ratings, GPT estimates, and fine-tuned models in same and different language.

## References

Alonso, M. Á., Díez, E., &amp; Fernandez, A. (2016). Subjective age-of-acquisition norms for 4,640 verbs in Spanish. *Behavior Research Methods, 48(4), 1337–1342.*

Alonso, M. A., Fernandez, A., &amp; Díez, E. (2015). Subjective age-of-acquisition norms for 7,039 Spanish words. *Behavior Research Methods, 47(1), 268–274.*

Botarleanu, R. M., Watanabe, M., Dascalu, M., Crossley, S. A., &amp; McNamara, D. S. (2024). Multilingual age of exposure 2.0. *International Journal of Artificial Intelligence in Education, 34(4), 1353–1377.*

Buechel, S., Rücker, S., &amp; Hahn, U. (2020). Learning and evaluating emotion lexicons for 91 languages. *arXiv preprint&nbsp;&nbsp;arXiv:2005.05672.*

Coltheart, M. (1981). The MRC psycholinguistic database. *The Quarterly Journal of Experimental Psychology Section A, 33(4), 497-505.*

Conde, J., González, M., Grandury, M., Reviriego, P., Martínez, G., &amp; Brysbaert, M. (2025a). Psycholinguistic Word Features: A New Approach for the Evaluation of LLMs Alignment with Humans. *In Proceedings of the Fourth Workshop on Generation, Evaluation and Metrics (GEM2), 8–17, Vienna, Austria. Association for Computational Linguistics.*

Conde, J., Martínez, G., Reviriego, P., Gao, Z., Liu, S., &amp; Lombardi, F. (2025b). Can ChatGPT learn to count letters? *Computer, 58(3), 96–99.*

Conde, J., Martínez, G., Grandury, M., Arriaga, C., Haro, J., Schroeder, S., ... &amp; Brysbaert, M. (2026). Updating the German Psycholinguistic Word Toolbox with AI-Generated Estimates of Concreteness, Valence, Arousal, Age of Acquisition, and Familiarity. *Journal of Cognition, 9(1), 9.*

Fu, T., Martínez, G., Conde, J., Arriaga, C., Reviriego, P., Qi, X., & Liu, S. (2026). Beyond Reproducibility: Token Probabilities Expose Large Language Model Nondeterminism. *arXiv preprint arXiv:2601.06118.*

Hollis, G., Westbury, C., &amp; Lefsrud, L. (2017). Extrapolating human judgments from skip-gram vector representations of word meaning. *Quarterly Journal of Experimental Psychology, 70(8), 1603–1619.*

Kuperman, V., Stadthagen-Gonzalez, H., &amp; Brysbaert, M. (2012). Age-of-acquisition ratings for 30,000 English words. *Behavior Research Methods, 44(4), 978–990.*

Martínez, G., Conde, J., Reviriego P. &amp; Brysbaert, M. (2025). *Generating lexical decision times with large language models: Dynamic use of megastudy data. Preprint*

Plisiecki, H., &amp; Sobieszek, A. (2024). Extrapolation of affective norms using transformer-based neural networks and its application to experimental stimuli selection. *Behavior Research Methods, 56, 4716–4731.*

Revelle, W., &amp; Revelle, M. W. (2015). Package ‘psych.’ *The comprehensive R archive network, 337(338), 161–165.*

Sendín, E., Conde, J., Reviriego, P., Haro, J., Ferré, P., Hinojosa, JA., Brysbaert, M. (2025). Combining the power of large language models with finetuning based on strategically collected human ratings: A case study about age-of-acquisition estimates of Spanish words. *Psicologica 46(2): e17563*

Solovyev, V., Islamov, M., &amp; Bayrasheva, V. (2022, November). Dictionary with the evaluation of positivity/negativity degree of the Russian words. *In International Conference on Speech and Computer (pp. 651–664). Cham: Springer International Publishing.*

Scott, G. G., Keitel, A., Becirspahic, M., Yao, B., &amp; Sereno, S. C. (2019). The Glasgow norms: Ratings of 5,500 words on nine scales. *Behavior Research Methods, 51(3), 1258–1270.*

Thompson, B., &amp; Lupyan, G. (2018). Automatic estimation of lexical concreteness in 77 languages. *In Proceedings of the Annual Meeting of the Cognitive Science Society (Vol. 40).*

Ward, N. (1998). Artificial intelligence and other approaches to speech understanding: Reflection on methodology. *Journal of Experimental &amp; Theoretical Artificial Intelligence, 10, 487–493.*

Wang, T., &amp; Xu, X. (2024). The good, the bad, and the ambivalent: Extrapolating affective values for 38,000+ Chinese words via a computational model. *Behavior Research Methods, 56(6), 5386–5405.*

1. This is the case, for instance, with ratings of arousal, where individuals give quite divergent ratings about which words they find arousing. [↑](#footnote-ref-1)
2. <https://huggingface.co/inference-endpoints/dedicated> [↑](#footnote-ref-2)
3. For example, in the GPT-4o tokenizer Integer numbers from 0 to 999 are represented using a single token. Instead decimal numbers are split into several tokens, for example 4.25 is represented by three tokens: ‘4’, ‘.’, ‘25’. Therefore, instead of using decimal numbers is better to use an extended range of integers. [↑](#footnote-ref-3)
4. Some universities and workplaces have interfaces to GPT with these functionalities. [↑](#footnote-ref-4)
5. <https://help.openai.com/en/articles/4936856-what-are-tokens-and-how-to-count-them> [↑](#footnote-ref-5)
6. <https://gptforwork.com/tools/openai-chatgpt-api-pricing-calculator> [↑](#footnote-ref-6)
